# Glucocorticoid-driven transcriptomes in human airway epithelial cells: commonalities, differences and functional insight from cell lines and primary cells

**DOI:** 10.1186/s12920-018-0467-2

**Published:** 2019-01-31

**Authors:** Mahmoud M. Mostafa, Christopher F. Rider, Suharsh Shah, Suzanne L. Traves, Paul M. K. Gordon, Anna Miller-Larsson, Richard Leigh, Robert Newton

**Affiliations:** 1 Airways Inflammation Research Group, Snyder Institute for Chronic Diseases, University of Calgary, Calgary, Alberta Canada; 2 Cardiovascular and Respiratory Sciences graduate program, Cumming School of Medicine, University of Calgary, Calgary, Alberta Canada; 3 Department of Medicine, Vancouver General Hospital, University of British Columbia, Vancouver, British Columbia Canada; 4 Centre for Health Genomics and Informatics, University of Calgary, Calgary, Alberta Canada; 5 Respiratory GMed, AstraZeneca Gothenburg, Mölndal, Sweden

**Keywords:** Glucocorticoid, Inhaled corticosteroid (ICS), Airway epithelium, Transactivation, Transrepression, Asthma

## Abstract

**Background:**

Glucocorticoids act on the glucocorticoid receptor (GR; NR3C1) to resolve inflammation and, as inhaled corticosteroids (ICS), are the cornerstone of treatment for asthma. However, reduced efficacy in severe disease or exacerbations indicates a need to improve ICS actions.

**Methods:**

Glucocorticoid-driven transcriptomes were compared using PrimeView microarrays between primary human bronchial epithelial (HBE) cells and the model cell lines, pulmonary type II A549 and bronchial epithelial BEAS-2B cells.

**Results:**

In BEAS-2B cells, budesonide induced (≥2-fold, *P* ≤ 0.05) or, in a more delayed fashion, repressed (≤0.5-fold, *P* ≤ 0.05) the expression of 63, 133, 240, and 257 or 15, 56, 236, and 344 mRNAs at 1, 2, 6, and 18 h, respectively. Within the early-induced mRNAs were multiple transcriptional activators and repressors, thereby providing mechanisms for the subsequent modulation of gene expression. Using the above criteria, 17 (BCL6, BIRC3, CEBPD, ERRFI1, FBXL16, FKBP5, GADD45B, IRS2, KLF9, PDK4, PER1, RGCC, RGS2, SEC14L2, SLC16A12, TFCP2L1, TSC22D3) induced and 8 (ARL4C, FLRT2, IER3, IL11, PLAUR, SEMA3A, SLC4A7, SOX9) repressed mRNAs were common between A549, BEAS-2B and HBE cells at 6 h. As absolute gene expression change showed greater commonality, lowering the cut-off (≥1.25 or ≤ 0.8-fold) within these groups produced 93 induced and 82 repressed genes in common. Since large changes in few mRNAs and/or small changes in many mRNAs may drive function, gene ontology (GO)/pathway analyses were performed using both stringency criteria. Budesonide-induced genes showed GO term enrichment for positive and negative regulation of transcription, signaling, proliferation, apoptosis, and movement, as well as FOXO and PI3K-Akt signaling pathways. Repressed genes were enriched for inflammatory signaling pathways (TNF, NF-κB) and GO terms for cytokine activity, chemotaxis and cell signaling. Reduced growth factor expression and effects on proliferation and apoptosis were highlighted.

**Conclusions:**

While glucocorticoids repress mRNAs associated with inflammation, prior induction of transcriptional activators and repressors may explain longer-term responses to these agents. Furthermore, positive and negative effects on signaling, proliferation, migration and apoptosis were revealed. Since many such gene expression changes occurred in human airways post-ICS inhalation, the effects observed in cell lines and primary HBE cells in vitro may be relevant to ICS in vivo.

**Electronic supplementary material:**

The online version of this article (10.1186/s12920-018-0467-2) contains supplementary material, which is available to authorized users.

## Background

Glucocorticoids are the most effective anti-inflammatory drugs currently available for the treatment of chronic inflammation [1]. They exert effects via the glucocorticoid receptor (GR; NR3C1), a ligand-activated transcription factor that is expressed in nearly all cells [2]. Under basal conditions, unliganded GR is retained as a multi-protein complex predominately in the cytoplasm. Upon ligand binding, GR undergoes conformational change, followed by alterations in the composition of the associated protein complex that ultimately promote shuttling of GR into the nucleus [3]. GR then coordinates a series of events that lead to the repression of inflammatory gene expression. Among the many possible forms of repression is transrepression in which a factor, here GR, may act in *trans* at another gene locus to elicit repression, for example, of inflammatory gene transcription. One form of GR transrepression, which is widely referred to as tethered, or tethering, transrepression, involves inhibition of DNA-bound inflammatory transcription factor activity via direct interactions with non-DNA bound GR [4, 5]. A second form of transrepression involves SUMOylated GR binding to *cis*-acting negative glucocorticoid response elements (nGREs) to inhibit gene transcription and is here referred to as *cis*-acting transrepression [6, 7]. However, being a transcriptional activator, GR may also bind to simple palindromic glucocorticoid response elements (GREs) or GRE half sites, and can interact with other transcription factors to enhance the expression of numerous genes [8–10]. This effect is referred to as transactivation and applies to multiple anti-inflammatory genes, as well as genes relevant to the developmental and metabolic effects of glucocorticoids [11–13]. While transactivation and transrepression by GR may occur concurrently [14], their relative weights in the overall repressive effect of glucocorticoids requires clarification [15, 16].

Despite profound anti-inflammatory effects, further exploitation of the clinical benefits of glucocorticoids is hindered by a knowledge gap in understanding the functional impact of the key glucocorticoid-modulated genes in relevant, target, or off-target, tissues. Identification of such genes may be problematic because the effects of glucocorticoids on gene expression are highly context-dependent and may show variable effects between different cell types, even those with similar lineage or tissues of origin [17]. Moreover, GR responses show considerable modulation depending on the presence of other co-stimuli, including culture conditions [18]. This increases variety in the GR-dependent transcriptome and confounds the identification of key gene expression features.

In asthma, glucocorticoids are extensively prescribed due to their anti-inflammatory properties and, as inhaled corticosteroids (ICS), they usually provide effective treatment in mild to moderate disease [1]. ICS act on multiple cell types, especially airway epithelial cells, to decrease the expression of cytokines, chemokines, adhesion molecules, and other inflammatory proteins in the airways. As airway epithelial cells lie at the interface between the environment and the host, and play critical roles in shaping inflammatory responses in the airways [19, 20], they are not only an initial ICS target, but have long been thought to be important to the anti-inflammatory effects of ICS [21, 22]. More recently, a central role for airway epithelial cells in the anti-inflammatory effects of glucocorticoids has been confirmed in a mouse model of allergic asthma [23]. Thus, understanding the effects of glucocorticoids on the airway epithelium is central to future attempts to improve ICS therapies. In this respect, the use of cultured airway epithelial cells for in vitro mechanistic investigations of airway inflammation and glucocorticoid responses is both widespread and necessary. Here, we report a comparative transcriptome analysis using submersion culture of 3 commonly used airway epithelial cell models, or variants; two epithelial cell lines, pulmonary type II A549 and bronchial epithelial BEAS-2B cells, and primary human bronchial epithelial (HBE) cells, in response to the clinically relevant ICS, budesonide. To link these in vitro data with the in vivo response to ICS in human airways, transcriptomic data from bronchial biopsies, obtained 6 h following budesonide inhalation in a prior placebo-controlled cross-over study [24], were combined into the current analysis. These gene- and function-based comparisons provide a platform for future functional interrogation of the key gene expression features of the glucocorticoid response in airway epithelial cells.

## Methods

### Cell culture

The A549 cell line (American Type Culture Collection; ATCC) was grown in Dulbecco’s modified Eagle’s medium (DMEM) (Invitrogen) supplemented with 10% fetal bovine serum (FBS) (Sigma-Aldrich) and 2 mM L-glutamine. The BEAS-2B cell line (ATCC) was grown in DMEM/F12 (Invitrogen) supplemented with 14 mM NaHCO_3_, 2 mM L-glutamine and 10% FBS. Primary human bronchial epithelial (HBE) cells were isolated from non-transplantable normal human lungs obtained through a tissue retrieval service at the International Institute for the Advancement of Medicine (Edison, NJ), as previously described [25, 26]. HBE cells were grown in submersion culture in bronchial epithelial cell growth medium (BEGM) (Lonza) containing all SingleQuots supplements (Lonza) except for hydrocortisone. All cells were incubated at 37 °C in 5% CO_2_. Cell lines were passaged when 90–95% confluent and then cultured either in 6 or 12-well plates, as appropriate. Prior to experiments, all cells were incubated overnight in basal media that were serum- and additive-free. Budesonide (AstraZeneca, Sweden) and dexamethasone (Steraloids, Newport, RI) were dissolved in dimethyl sulphoxide (DMSO) (Sigma-Aldrich) as stocks of 10 mM. Final DMSO concentrations on cells were ≤ 0.1%.

### RNA isolation, cDNA synthesis and SYBR green real-time qPCR

Total RNA was extracted using the NucleoSpin RNA (D-Mark Biosciences) and quantified with NanoDrop (Thermo). Then, cDNA was prepared using a qScript cDNA synthesis kit (Quantabio) from 0.5 μg of RNA, before being diluted 1:4 in PCR quality water. PCR was carried out on 2.5 μl of cDNA using Fast SYBR Green Master Mix (Applied Biosystems). A StepOne Plus PCR system (Applied Biosystems) was utilized for real-time PCR analysis. Relative cDNA concentrations were obtained from standard curves generated by serial dilution of cDNA obtained from total lung RNA and analyzed at the same time as experimental samples. The conditions for amplifications were: 50 °C for 2 min, 95 °C for 10 min, then 40 cycles of 95 °C for 15 s, 60 °C for 1 min. Primer pairs specific to genes of interest were utilized (Additional file 1). For genes with more than one splice variant, primers were designed to pick up all variants. All primers were designed using Primer BLAST (NCBI) and were synthesized by the DNA synthesis lab at the University of Calgary. Primer specificity was determined using dissociation (melt) curve analysis: 95 °C for 15 s, 60 °C for 20 s followed by ramping to 95 °C with florescence measurement every 2.5 degrees. A single peak in the change of fluorescence with temperature indicates acceptable specificity of primers.

### Microarray analysis

Following quality control (Agilent RNA 6000 Nano LabChips), RNA samples were subjected to PrimeView microarray (Affymetrix) preparation and analysis per the manufacturer’s specifications and scanned using a GeneChip 3000 scanner (Affymetrix). Robust multiarray averaging (RMA), quantile normalization, and median polishing on logged probe set intensity values were performed using Affymetrix Transcriptome Analysis Console (TAC) software. Fold change of the probe set intensity values to the matched untreated controls were calculated and one-way analysis of variance (ANOVA) was performed using Transcriptome Analysis Console (TAC) software (Affymetrix). Normalization and probe set analysis were performed for each sample type independently. Where genes have multiple probe sets, only probe sets showing the greatest overall level of change were retained for subsequent analyses. For heatmap presentation, log_2_ probe emission intensity of each replicate was obtained from CEL files, then the data from treated samples and their corresponding controls were zero-meaned. Such normalization unified the scale of gene expression change without negating the magnitude of change.

### Bronchial biopsy data

We recently described the gene expression changes due to budesonide inhalation in the human lung, where bronchial biopsy samples taken from normal individuals 6 h after a single inhalation of budesonide (1600 μg) or placebo were analysed using Affymetrix PrimeView microarray [24]. The gene expression data from that study was used in the current analysis for comparative purposes with transcriptome data from primary cells and cell-lines.

### Functional annotation analysis

In downstream gene ontology (GO) and pathway analyses of microarray data, the Database for Annotation, Visualization, and Integrated Discovery (DAVID) v6.8 was used [27]. Functional annotation charts for molecular functions (MF) and biological processes (BP) GO terms as well as KEGG pathway terms were obtained. DAVID default cut-off (enrichment *P* value (EASE score) ≤ 0.1) was used to define enriched pathways. Additional, more conservative, criteria were considered in some analyses, such as limiting the output to terms associated with at least 5 genes instead the default 2-genes cut-off. The multiple testing correction of enrichment *P* values (Benjamini) were also obtained to highlight robustly enriched terms. Ingenuity Pathway Analysis software IPA® (Qiagen) was used to estimate the associated pathways with the changes in gene expression as well as activation/inhibition scores of such pathways.

### Graphical presentation

GraphPad Prism version 6 software (GraphPad Software Inc., La Jolla, CA) was used to produce dose-response curves, scatter plots, and correlation diagrams. The R packages; “*pheatmap*” was used to produce heatmaps, “*VennDiagram*” was used to produce Venn diagrams that illustrate the overlap between 3 groups, and “*UpSetR*” was used to illustrate the overlap between 4 groups.

## Results

### Maximally effective glucocorticoid concentrations in epithelial cell lines and primary human bronchial epithelial cells

In prior studies, budesonide and dexamethasone elicited maximal responses at 100–300 nM or 300–1000 nM, respectively, on a simple 2 × GRE-driven luciferase reporter that was stably transfected into bronchial epithelial, BEAS-2B, or pulmonary type II epithelial, A549, cells [28, 29]. Similar maximally effective concentrations were also achieved for multiple glucocorticoid-induced mRNAs, including DUSP1, FKBP5, RGS2, and TSC22D3 with budesonide and dexamethasone in A549 and/or BEAS-2B cells [30–32]. Thus, budesonide at 300 nM and dexamethasone at 1 μM ensures GR saturation to produce maximal responses and these concentrations were selected for transcriptome analyses. Primary HBE cells were treated with increasing concentrations of budesonide, and mRNA expression of FKBP5, RGS2, TLR2 and TSC22D3, genes previously documented as being glucocorticoid-induced in these cells [24], was examined (Additional file 2). Log EC_50_ values were − 8.3, − 8.0, − 8.1, and − 8.3 for FKBP5, RGS2, TLR2 and TSC22D3, respectively. Maximal mRNA induction was achieved with 100 nM budesonide and this was used in the subsequent transcriptome analyses.

### Kinetics of budesonide-modulated gene expression in BEAS-2B cells

**Fig. 1:**
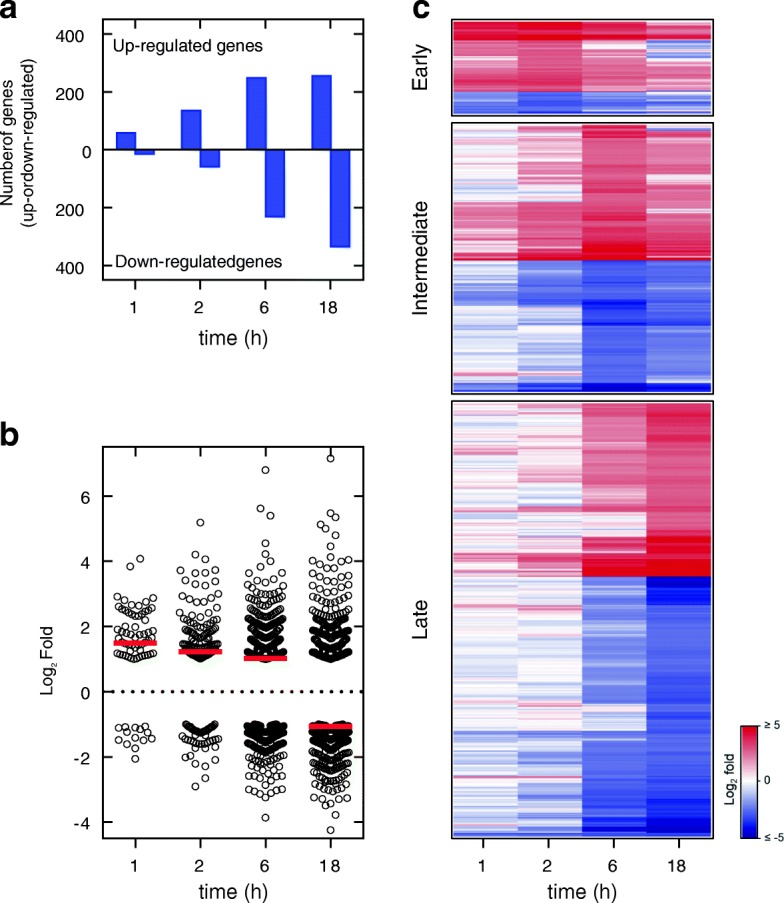
Kinetic profile of budesonide-modulated gene expression in BEAS-2B cells. BEAS-2B cells were either not treated or treated with budesonide (300 nM) prior to harvesting at 1, 2, 6, and 18 h post-treatment. RNA was prepared, and microarray analysis performed using Affymetrix Primeview arrays. Probe set intensities (*N* = 4) from different samples were normalized using the Robust Multi-Array Average (RMA) method. Comparing the probe set intensities of the treated samples against their time-matched untreated controls identified 820 genes that were induced ≥2 fold (*P* ≤ 0.05) or repressed ≤0.5 fold (*P* ≤ 0.05) by budesonide at any time-point (Additional file 3). **a**. Bar graph illustrating the number of genes meeting the induction or repression criteria at each time-point. **b**. Scatter plot showing log_2_ fold change for genes that were induced or repressed at each time. The overall mean of log_2_ fold change is depicted as a solid red line. **c**. Genes were grouped based on the time point that showed the greatest absolute log_2_ fold change into; early (1–2 h; 93 genes), intermediate (6 h; 263 genes) or late (18 h; 463 genes). Heat maps show the relative expression of genes within each group. Data are represented as log_2_ fold change relative to the corresponding untreated controls

To examine the temporal nature of the gene expression changes produced by glucocorticoids, BEAS-2B cells were treated with budesonide (300 nM) for 1, 2, 6 and 18 h prior to RNA extraction and analysis using Affymetrix Primeview microarrays. Probe set intensities from different samples were normalized using the robust multi-array average (RMA) method. Gene expression changes were expressed as fold of budesonide-treated compared to untreated control at each time point. There were 63, 133, 240 and 257 genes induced ≥2 fold (*P* ≤ 0.05) at 1, 2, 6, and 18 h, respectively, and 15, 56, 236, and 344 genes repressed ≤0.5 fold (*P* ≤ 0.05), respectively (Fig. 1a; Additional file 3). While the number of genes induced by budesonide at the early times (1 and 2 h) markedly outnumbered the repressed genes, at later times, in particular 18 h, repressed genes were found to predominate (Fig. 1a). This shift from induction towards repression was also evident in the overall average log_2_ fold change for all the genes that were significantly modulated by budesonide at each time point (Fig. 1b).

The 820 genes showing significantly enhanced (≥2 fold, *P* ≤ 0.05), or repressed (≤0.5 fold, *P* ≤ 0.05), expression at any time were placed into three groups; early (1–2 h, 93 genes), intermediate (6 h, 263 genes) and late (18 h, 463 genes) (Additional file 3), based on the time of greatest absolute log_2_ fold change, and heat maps were generated (Fig. 1c). Notably, 51% of the early genes and 37% of the late genes revealed differential expression that also met the above inclusion criteria at 6 h. Furthermore, if less stringent cut-off criteria (≥1.25 fold or ≤ 0.8 fold, *P* ≤ 0.05) were applied then 86% of the early genes and 85% of the late genes were identified as differentially expressed at 6 h (Additional file 3). Thus, 6 h was selected as being broadly representative for transcriptome analyses to compare primary cells and cell lines.

### Gene expression changes in response to different glucocorticoids

**Fig. 2:**
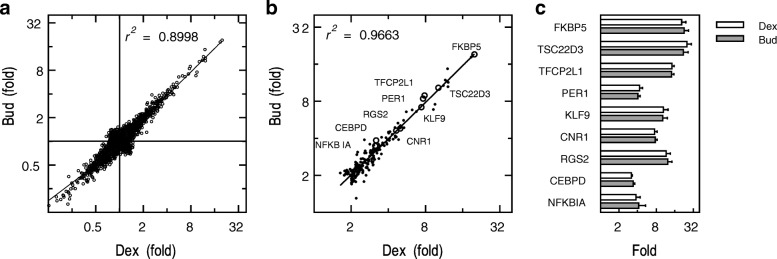
Effect of budesonide and dexamethasone on gene expression in A549 cells. A549 cells were either not treated or treated with 300 nM budesonide (Bud) or 1 μM dexamethasone (Dex). After 6 h, RNA was prepared, and microarray analysis performed using Affymetrix Primeview arrays. Probe set intensities (*N* = 3) from the different samples were normalized using the robust multi-array average (RMA) method and used to calculate the fold change compared to untreated (Additional file 4). **a**. Fold change, compared to untreated, for the dexamethasone and budesonide treatments, is plotted for each gene on the x and y axes, respectively. **b**. The relationship between the fold change for each treatment is shown in respect of all genes induced ≥2 fold (*P* ≤ 0.05) by either budesonide or dexamethasone treatment. Genes selected for qPCR validation are shown as circles (○) labelled with their gene symbol. **c**. A549 cells were either not treated or treated with 300 nM budesonide or 1 μM dexamethasone. After 6 h, RNA was extracted, and qPCR performed for the indicated genes and GAPDH. Data (*N =* 6), normalized to GAPDH, were expressed as fold relative to untreated control and are plotted as means ± SE

While the effects of inhaled budesonide on gene expression in the human airways occurring 6 h post-inhalation were reported [24], in vitro cell-based studies generally use dexamethasone for analysis. As different ligands may produce variable, but GR-dependent, effects on gene expression [12, 17], plus may show different off-target effects, a comparative transcriptome analysis was performed using dexamethasone and budesonide. A549 cells were not treated or treated with either budesonide (300 nM) or dexamethasone (1 μM) for 6 h prior to microarray analysis. Budesonide and dexamethasone significantly induced (≥2 fold, *P* ≤ 0.05) 187 and 188 genes, respectively, and repressed (≤0.5 fold, *P* ≤ 0.05) 106 and 102 genes, respectively (Additional file 4). Both ligands elicited highly correlated gene expression profiles with *R*^*2*^ of 0.8998 when comparing the fold-change due to glucocorticoid treatment for all genes (induced and repressed) and 0.9663 in respect of the induced genes (≥2 fold, *P* ≤ 0.05) (Fig. 2 a, b; Additional file 4). Furthermore, using ANOVA to compare the probe set intensity values produced by budesonide and dexamethasone treatment showed no significant differences for any gene. This effect was validated by qPCR for 9 glucocorticoid-induced genes that revealed a range of induction levels in the microarray data (Fig. 2b, c). In each case, the fold induction for each gene was statistically indistinguishable between dexamethasone and budesonide (Fig. 2c). These data indicate that the response of A549 cells to a maximally effective concentration of dexamethasone or budesonide was equivalent.

### Budesonide-induced genes in airway epithelial cells

**Fig. 3:**
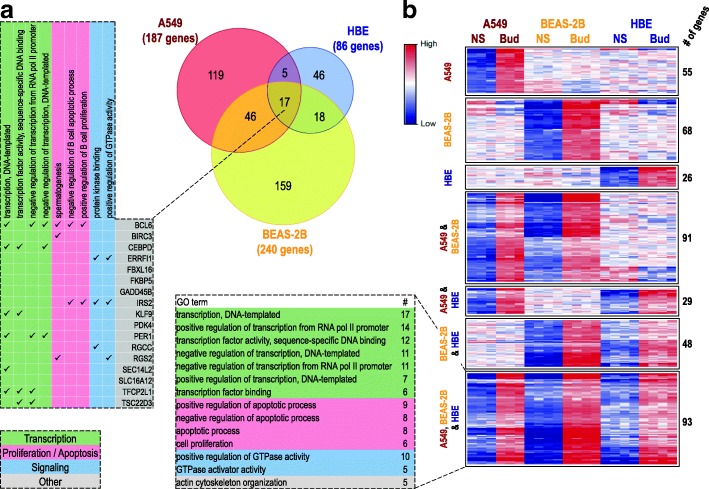
Genes induced by budesonide in A549, BEAS-2B and primary human bronchial epithelial (HBE) cells. A549 (*N =* 3), BEAS-2B (*N =* 4), and primary HBE (*N =* 4 donors) cells were not treated or treated with budesonide (300 nM for A549 and BEAS-2B, 100 nM for HBE) for 6 h. RNA was prepared, and microarray analysis performed using Affymetrix Primeview arrays. Probe set intensities from the different samples were normalized using the robust multi-array average (RMA) method. Comparing the probe set intensities of the treated samples against their untreated controls identified 410 genes showing significant induction ≥2 fold (*P* ≤ 0.05) compared to untreated control in any of the cell variants (Additional file 5). **a**. The genes meeting these criteria in each cell variant were used to produce a Venn diagram to illustrate commonality between the three cell variants. The 17 genes induced in common were subjected to GO analysis in DAVID and are shown along with GO terms showing significant enrichment (EASE score ≤ 0.1) with genes from this list. Genes representing each term are indicated. GO terms are categorized into 3 primary groups (transcription, proliferation/apoptosis or signaling) as indicated. **b**. The 410 genes were then grouped as defined by the lower stringency, ≥ 1.25 fold, cut-off (Additional file 5). The genes within each group are listed in Additional file 5. Heat maps were constructed for each grouping. Data are presented as zero-mean log_2_ intensity, centered around the mean of untreated (not stimulated; NS) and treated for each gene in each cell variant. Following GO analysis in DAVID, GO terms, showing significant enrichment (EASE score ≤ 0.1) and representing at least 5 genes of the 93 genes induced in common, are listed. The number of genes associated with each term are indicated. The GO terms were then categorized into 3 primary groups (transcription, proliferation/apoptosis or signaling), or other, as shown

A549, BEAS-2B and primary HBE cells were either not treated or treated with a maximally effective concentration of budesonide for 6 h followed by transcriptome analysis using Affymetrix PrimeView microarrays. Expressing gene expression change as fold of untreated produced 187, 240 and 86 genes that were induced ≥2 fold (*P* ≤ 0.05) in A549, BEAS-2B, and HBE cells, respectively (Fig. 3a). While this gave an overall total of 410 genes induced by budesonide (Additional file 5), application of a ≥ 2 fold (*P* ≤ 0.05) cut-off revealed only 17 genes in common between the 3 epithelial cell variants (Fig. 3a). However, a further 5, 18 and 46 genes were common between A549/HBE, BEAS-2B/HBE and A549/BEAS-2B cells, respectively, with 119, 159 and 46 genes uniquely induced (≥2 fold, *P* ≤ 0.05) by A549, BEAS-2B and HBE cells, respectively.

Initial gene ontology (GO) analysis using Database for Annotation, Visualization, and Integrated Discovery (DAVID) showed the list of 17 genes in common to be enriched (EASE score ≤ 0.1; DAVID’s modified Fisher’s exact test default [27]) in 9 GO terms for molecular function or molecular process that can be generically categorized as: transcriptional control (BCL6, CEBPD, KLF9, PER1, SEC14L2, TFCP2L1, and TSC22D3), apoptosis/proliferation (BCL6, BIRC3, IRS2, and RGS2), or signaling (ERRFI1, IRS2, RGCC, and RGS2) (Fig. 3a). Although a number of these genes are likely to exert a positive influence on transcription, others are clearly negative regulators of transcription, as is indicated by the GO terms: “negative regulation of transcription from RNA pol II promoter” and “negative regulation of transcription, DNA-templated”. Likewise, other GO terms indicate positive effects on proliferation and negative effects on apoptosis. Of the four genes associated with GO terms for signaling, one of these, RGS2, a positive regulator of GTPase activity, promotes inactivation of signaling from many pro-asthma G-protein coupled receptors (GPCRs) and is implicated in the therapeutic benefits of glucocorticoids in the airways [33–36]. ERRFI1 and IRS2 relate to tyrosine kinase-linked and insulin signaling, while the fourth signaling gene, RGCC, is a regulator of the cell cycle and is therefore also likely to operate alongside other genes involved in proliferation and/or apoptosis. Finally, the presence of PDK4, a pyruvate dehydrogenase kinase that inactivates the pyruvate dehydrogenase complex to regulate glucose metabolism, speaks to the metabolic effects of glucocorticoids.

Hierarchical clustering combined with heat map generation, not only confirmed extensive cell variant-dependent differences in gene expression, but also revealed a considerably greater level of commonality than was evident from the application of a simple ≥2 fold cut-off (data not shown). To capture this, a less stringent, ≥1.25 fold, cut-off for inducibility in any cell variant was applied to the 410 genes without further inclusion of additional genes to produce seven expression groups that are displayed as heat maps (Fig. 3b). Such threshold effectively captures genuine gene expression changes as: *i)* 91, 98 and 72% of all genes showing ≥1.25 fold induction in A549, BEAS-2B or HBE cells, respectively, were significantly (*P* ≥ 0.05) induced; and, *ii)* these 410 genes all showed significant ≥2 fold inducibility in at least one other epithelial cell variant (Additional file 5). The largest of these groups, contains 93 genes that are in common across A549, BEAS-2B and primary HBE cells (Fig. 3b; Additional file 5). While the next largest group (91 genes) confirms considerable additional commonality between A549 and BEAS-2B cells, the heat map reveals some genes that respond in an opposite manner in HBE cells (Fig. 3b). Likewise, 29 and 48 genes in A549 or BEAS-2B cells, respectively, showed similar responses in the HBE cells. Finally, 55, 68 and 26 genes, showed A549-, BEAS-2B, or HBE-specific responses, respectively.

Using DAVID to identify GO terms for biological process and molecular function showed that multiple terms for transcriptional regulation and control were significantly enriched (EASE score ≤ 0.1) with the list of 93 genes induced in common (Fig. 3b). Thus, 30% (28 genes) of these genes were associated with GO terms, including “positive regulation of transcription from RNA polymerase II promoter”, and “negative regulation of transcription, DNA-templated”. Many transcription factors, including CEBPD, FOXO3, KLF4, KLF9, TFCP2L1, and ZBTB16, as well as regulators of signaling, including BCL6, CDKN1C, and PIK3R1, and chromatin remodelling factors, such as CITED2, may all produce transcriptional effects and are readily identifiable within this gene list. Importantly, the number of genes, 15%, associated with “positive regulation of transcription from RNA polymerase II promoter” and, 11%, associated with “negative regulation of transcription, DNA-templated”, reflects the two core activities, namely activation and repression of gene expression by GR [11, 16]. In addition, 20 genes were associated with terms related to cellular apoptosis and proliferation and 11 genes were associated with signaling terms, specifically those related to modulation of GTPase activity.

### Validation of budesonide-induced gene expression

The array intensity values and fold change for the genes within each of the seven expression groups in Fig. 3b were summarized (Additional file 6 a, b) and 52 genes representative of each group were subjected to qPCR (Additional file 6 c). Comparing fold change obtained from the microarray analysis with that from qPCR showed most genes cluster around the line of unity for each cell variant (Additional file 6 d). Among genes in the array data that were induced ≥1.25 fold by budesonide, the percentage showing ≥1.25 fold induction by qPCR was 95, 100, and 92% in A549, BEAS-2B and HBE cells, respectively (Additional file 6 c), i.e. on average, the array data was confirmed by qPCR in greater than 95% of instances. Furthermore, for most of these genes, this was at a level where the fold induction detected by qPCR also exceeded 2 fold (85, 82, and 67%, for A549, BEAS-2B and HBE cells, respectively). As a consequence, the increased budesonide fold-inducibility shown by qPCR for 15 genes indicated a higher degree of commonality between the cell variants than was previously evident from the microarray data (Additional file 6 c). Nevertheless, qPCR failed to confirm the placement of 4 genes (Additional file 6 c). For NCOA3, NFKBIA, and TGFBR2, this can be attributed to the borderline induction by budesonide (~ 1.25 fold) in the array data (Additional file 5), whereas for PHACTR3, there was a possible failure of the microarray probe set to detect the annotated gene. As is shown by deviation in the best fit line from the line of unity (Additional file 6 d), the microarray analysis generally under-represented fold induction relative to qPCR. Since genes showing pronounced differences in fold induction by qPCR compared to the array (qPCR fold/array fold ≥2) had average basal C_T_ values of 32.0, 32.8, and 29.9 in A549, BEAS-2B and HBE cells, respectively, underrepresentation by the microarray may be a simple consequence of low basal expression. Nevertheless, these data clearly show that reliance on a strict 2 fold cut-off (*P* ≤ 0.05) in analyzing microarray data under-reports budesonide-induced gene expression.

### Comparative analysis of GO terms associated with budesonide-induced genes

**Fig. 4:**
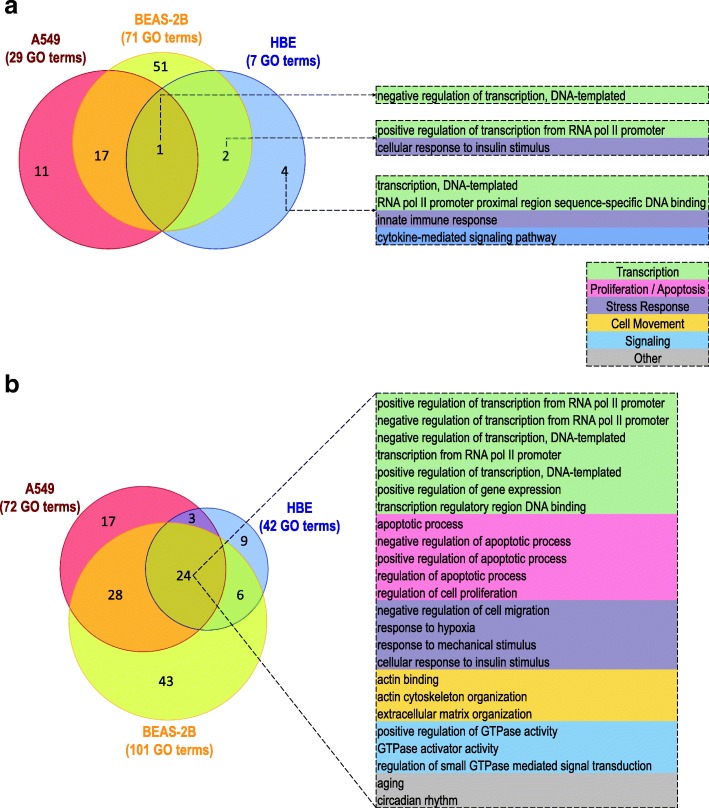
Commonalities between GO terms enriched with budesonide-induced genes in A549, BEAS-2B and HBE cells. **a**. Budesonide-induced genes (≥ 2 fold, *P* ≤ 0.05; Additional file 5) in A549 (187 genes), BEAS-2B (240 genes), and HBE (86 genes) were each subjected to GO analysis using DAVID. Biological process and molecular function terms showing significant enrichment (EASE score ≤ 0.1) with each list, and also represented by at least 5 genes, were obtained (Additional file 7). The number of GO terms enriched in each cell variant is indicated and the Venn diagram illustrates the overlap in terms between each cell variant. Terms showing enrichment in HBE cells are provided to the right and are categorized (color coded) according to main function. **b**. This analysis was repeated using the lower stringency budesonide-induced gene lists, as defined in Fig. 3b, and the resultant GO terms are listed in Additional file 8. Total numbers of enriched GO terms for each cell variant are indicated and the Venn diagram illustrates overlap between terms in the 3 cell variants. To the right are the 24 GO terms that were enriched in common for all cell variants. These were categorized as in panel **a**

The gene lists of ≥2 fold (*P* ≤ 0.05) budesonide-induced genes in each epithelial cell variant from Fig. 3a were entered into the DAVID functional annotation tool to identify GO terms for biological process and molecular function that were significantly enriched (EASE score ≤ 0.1) with at least 5 budesonide-induced genes in each cell variant. This produced 29, 71 and 7 GO terms in A549, BEAS-2B, and HBE cells, respectively (Fig. 4a, Additional file 7), of which only the term “negative regulation of transcription, DNA-templated” was common to all cell variants. Terms concerned with positive transcription and insulin signaling were also conserved between BEAS-2B and HBE cells, with additional terms for transcription, innate immunity, and cytokine signaling being restricted to HBE cells (Fig. 4a).

The lower stringency budesonide-induced gene lists, as defined in Fig. 3b, in respect of each cell variant were used to further assess common and unique functional impacts of budesonide treatment. As above, gene lists were analysed using the DAVID functional annotation tool to identify biological processes and molecular function terms showing enrichment (EASE score ≤ 0.1) with at least 5 of the budesonide-induced genes in each cell variant. This produced 72, 101, and 42 GO terms associated with the budesonide-induced genes in A549, BEAS-2B, and HBE cells, respectively (Fig. 4b, Additional file 8). This revealed 24 terms that were common to the gene lists from all cell variants. Categorizing these GO terms based on the parent classification showed; 7 linked to transcriptional regulation, 5 categorized as proliferation/apoptosis, 4 as stress response, 3 as cell movement, and 3 as signaling (Fig. 4b). In addition, the terms “cytokine-mediated signaling pathway”, “regulation of cell cycle”, and “positive regulation of angiogenesis” were common between HBE and A549 cells. A further 6 GO terms common to BEAS-2B and HBE cells included: terms for transcription (“transcription factor activity, sequence-specific DNA binding”); two terms (“negative regulation of cells growth”, “heart development”) involved in cell growth and development; and, two signaling terms (“phosphatidylinositol phosphorylation”, “negative regulation of canonical Wnt signaling pathway”). Finally, the nine GO terms that were restricted to the HBE cells, not only included additional terms for transcriptional control, signaling, proliferation and development, but also wound healing and circadian regulation (Additional file 8).

### Budesonide-repressed genes in airway epithelial cells

**Fig. 5:**
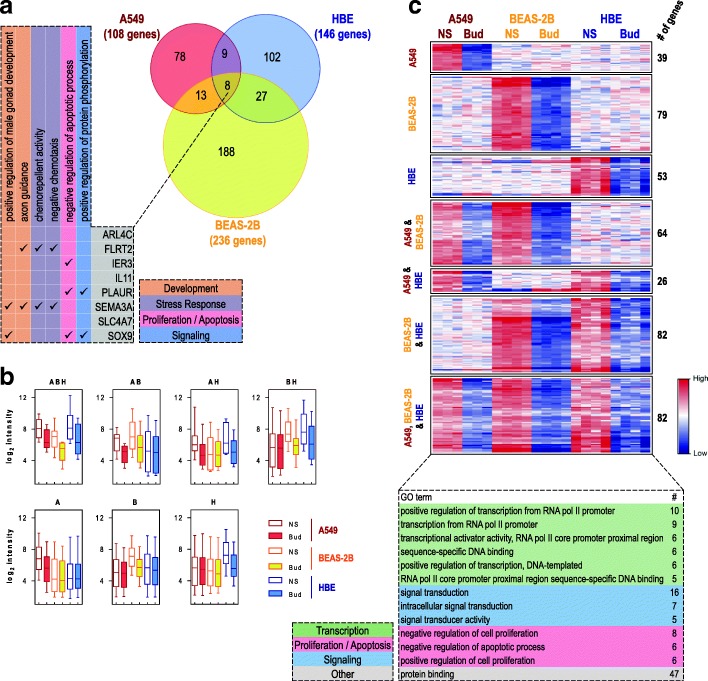
Genes repressed by budesonide in A549, BEAS-2B and primary HBE cells. As detailed for Fig. 3, microarray analysis identified 425 genes as significantly repressed by budesonide (≤0.5 fold, *P* ≤ 0.05) in any of the three cell variants (Additional file 9). **a**. Genes meeting these criteria were used to produce a Venn diagram to illustrate commonality between the three cell variants. The 8 genes repressed in common are listed and, following analysis in DAVID, GO terms showing significant enrichment (EASE score ≤ 0.1) with the 8 genes from this list are shown. Genes representing each GO term are indicated. Terms are categorized into 4 functional groups, as indicated. **b**. Genes within each group (A = A549, B = BEAS-2B and H = HBE), according to the Venn diagram in **a**, are summarized by log_2_ intensity. Data are color-coded (Red = A549, yellow/orange = BEAS-2B, Blue = HBE) where open bars represent untreated (NS) and solid bars are budesonide (Bud) treatments. The box defines the upper and lower quartiles, and the line inside represents the median. Whiskers represent the 5th and 95th percentiles. **c**. The 425 genes were then grouped using the lower stringency, ≤0.8 fold, cut-off (Additional file 9). Heat maps were constructed for each of the groups. Data are presented as zero-mean log_2_ intensity, centered around the mean of untreated (NS) and treated for each gene in each cell variant. GO terms, showing enrichment (EASE score ≤ 0.1) and represented by at least 5 genes in the 82 gene repressed in common group, are listed. The number of genes associated with each term are indicated. Terms were then categorized into functional groups, as indicated

Microarray analysis of budesonide-treated cells identified 108, 236, and 146 genes that were repressed ≤0.5 fold (*P* ≤ 0.05) by budesonide in A549, BEAS-2B, and HBE cells, respectively, giving a total of 425 genes repressed in any of the cell variants (Fig. 5, Additional file 9). Using these criteria, only 8 genes were commonly repressed in all three cell variants (Fig. 5a). Initial GO analysis using DAVID returned 6 GO terms showing enrichment for these commonly repressed genes. These can be categorized as; development (2 terms represented by FLRT2, SEMA3A and SOX9), stress response (2 terms represented by FLRT2, and SEMA3A), a proliferation/apoptosis term (IER3, PLAUR, and SOX9), and a signaling term (PLAUR and SOX9) (Fig. 5a).

Examination of the probe set intensities for the gene groups showing either repression, or no repression, by budesonide in the three cell variants revealed repression for gene groups with higher basal expression, but no repression for groups with lower basal expression (Fig. 5b). This effect is likely to occur as the gene expression level approaches the detection limit for a given probe set, and therefore cannot show further repression. Thus the detection of repression depends not only on the ability of the glucocorticoid to target the gene for repression, but also on the expression level of that gene in that cell variant.

As was observed for the induced genes, hierarchical clustering and the generation of a heat map for the 425 repressed genes in any variant revealed a considerably greater level of commonality than was suggested by the application of a simple ≤0.5 fold (*P* ≤ 0.05) criterion (data not shown). To capture this commonality, while mirroring the less stringent ≥1.25 fold (i.e. 0.3219 as log_2_) cut-off that was applied to the induced genes, a symmetrical − 0.3219 log_2_ fold, i.e. ≤ 0.8 fold, cut-off was applied to the 425-repressed gene list without further inclusion of additional genes (Additional file 9). This produced 7 gene groupings that are displayed as heat maps to illustrate the gene expression pattern changes in each cell variant (Fig. 5c). Accordingly, 211, 307, and 243 genes were repressed in A549, BEAS-2B and HBE cells with 80, 97, and 79%, respectively, showing *P* values ≤0.05. Of these, 82 genes were repressed in common by budesonide in all three cell variants. There was considerable additional commonality between BEAS-2B and HBE cells (82 genes), and between A549 and BEAS-2B cells (64 genes), while only 26 genes were repressed in common between A549 and HBE cells. Finally, 39, 79, and 53 genes showed cell-variant specific repression in A549, BEAS-2B and HBE cells, respectively (Fig. 5c).

GO analysis of those genes repressed in common (82 genes) revealed that 25% (21 genes) were associated with signaling terms, 18% (15 genes) were associated with proliferation/apoptosis terms, and 16% (13 genes) were associated with transcriptional regulation terms. Accordingly, a number of cytokine/chemokine, or similar, genes (CXCL1, CXCL8, IL11, SEMA3A, TNFSF9), growth factor/growth factor modulatory genes (VEGFC, FGFBP1), and transcription factors/transcriptional regulators (CREB5, E2F7, FOXC1, GRHL1, NFAT5, NR3C1, SOX9, TFAP2A) are readily apparent (Additional file 9). The presence of NR3C1, i.e. GR, is consistent with the known glucocorticoid-dependent downregulation of GR expression [37, 38].

### Comparative analysis of GO terms associated with budesonide-repressed genes

**Fig. 6:**
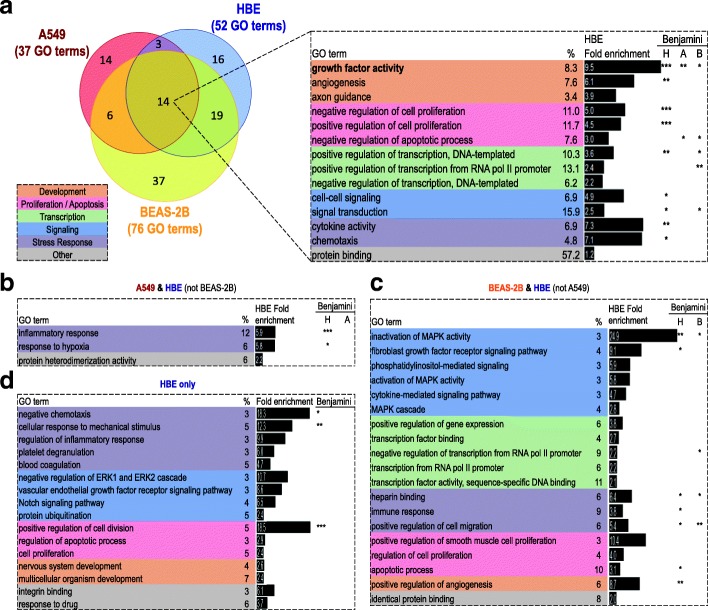
Commonalities between GO terms enriched with the budesonide-repressed (≤0.5 fold, *P* ≤ 0.05) genes in A549, BEAS-2B and HBE cells. Budesonide-repressed genes (≤0.5 fold, *P* ≤ 0.05; Additional file 9) in each of A549 (108 genes), BEAS-2B (236 genes), and HBE (146 genes) were subjected to GO analysis using DAVID. Biological process and molecular function terms showing significant enrichment (EASE score ≤ 0.1) with each list, and represented by at least 5 genes, were obtained (Additional file 10). **a**. The number of GO terms enriched in the gene lists from each cell variant is indicated and overlaps are shown in the Venn diagram. The 14 GO terms enriched in common for all cell variants are shown. **b**. Three GO terms enriched in common in A549 and HBE cells, but not in BEAS-2B cells. **c**. Nineteen GO terms enriched in common in BEAS-2B and HBE cells, but not in A549 cells. **d**. Sixteen GO terms uniquely enriched in HBE cells. Each box (dotted lines in panels **a** – **d**) also depicts the percentage of the genes associated with each term within the total number of genes repressed in HBE cells and the fold enrichment of each term in the HBE cell list. Significance, following correction for family-wide false discovery, in A549 (A), BEAS-2B (B), and HBE (H) cells, is indicated where: Benjamini ≤0.05 (*), Benjamini ≤0.01 (**), or Benjamini ≤0.001 (***)

The budesonide-repressed gene (≤ 0.5 fold, *P* ≤ 0.05) list for each cell variant was subjected to GO analysis using DAVID. This produced 37, 76 and 52 GO terms, each represented by at least 5 genes, and which showed significant enrichment (EASE score ≤ 0.1) for the repressed genes in each of A549, BEAS-2B, and HBE cells, respectively (Fig. 6, Additional file 10). Of these, 14, including the generic term “protein binding”, were common between A549, BEAS-2B and HBE cells and can be categorized as development (3 terms), proliferation/apoptosis (3 terms), transcription (3 terms), signaling (2 terms), and stress response (2 terms) (Fig. 6a). With terms including “cell-cell signaling”, “signal transduction”, “cytokine activity”, and “chemotaxis”, this correlates well with the repressive effects of glucocorticoids on inflammation and inflammatory gene expression. This is also supported by terms for the positive regulation of transcription being associated with the repressed gene lists for each cell variant. However, the GO term “negative regulation of transcription, DNA templated” was also associated with the repressed gene list and implies some loss of transcriptional repression. Similarly, GO terms relating to both positive and negative effects on proliferation are apparent. Of note was that many of these GO terms, for example “signal transduction”, “cell-cell signaling”, “chemotaxis”, “cytokine activity” in HBE cells, as well as multiple terms for cell proliferation and apoptosis and transcriptional control, were more robustly enriched (Benjamini ≤0.05) (Fig. 6a). Many similar enrichments were also apparent in A549 and BEAS-2B cells, and the term “growth factor activity” showed 9.47 fold enrichment in the HBE repressed gene-list and was also more significantly enriched (Benjamini ≤0.05) in both A549 and BEAS-2B cells (Fig. 6a).

The above concepts are further reinforced by consideration of the enriched (EASE score ≤ 0.1) GO terms in the A549/HBE and BEAS-2B/HBE overlaps (Fig. 6b, c). For example, within the 3 GO terms in the A549/HBE overlap there are GO terms for “inflammatory response” and “response to hypoxia”, i.e. stress responses, and both these terms also reveal robust (Benjamini ≤0.05) 5–6 fold enrichments in the HBE gene list (Fig. 6b). Likewise, within the 19 GO terms in the BEAS-2B/HBE overlap, numerous terms are highly enriched and also show robust (Benjamini ≤0.05) enrichments (Fig. 6c). Examples include, “positive regulation of angiogenesis” and “fibroblast growth factor receptor signaling pathway”, which are enriched 8.65 and 9.10 fold, respectively, in the repressed HBE cell gene list. Other enriched terms in the HBE cells include; “apoptotic process”, “immune response”, and “positive regulation of cell migration”. Additionally, and contrary to expectation, “inactivation of MAPK activity” is strongly enriched in the repressed lists in both the HBE and BEAS-2B cells. The genes (in BEAS-2Bs: DUSP14, DUSP4, DUSP8, RGS3, RGS4; or, in HBE cells: DUSP4, DUSP6, RGS3, SPRED1, SPRED2), which associate with this GO term, certainly show functions that would be predicted to reduce inflammatory signaling cascades, including MAPKs. Therefore, the observed repression of these genes should promote MAPK activation, yet multiple studies clearly show that MAPK activity is reduced by glucocorticoids in these cell variants [32, 39]. Finally, various GO terms, for example “positive regulation of cell division”, “cellular response to mechanical stimulus” and “negative chemotaxis”, were robustly enriched in the repressed gene list in HBE cells, but may not be modelled in the two cell lines (Fig. 6d).

Using the lower stringency cut-off (≤0.8 fold), as defined in Fig. 5c for repressed genes in each cell variant, identified 70, 114, and 88 enriched (EASE score ≤ 0.1) GO terms in A549, BEAS-2B, and HBE cells, respectively (Additional file 11). Plotting as a Venn diagram showed 41 GO terms were common across the repressed genes from all three variants (Additional file 12). In addition to three generic terms (“protein binding”, “protein heterodimerization activity” and “response to drug”), these terms were categorized as transcriptional regulation (11 terms), stress response, including inflammatory response (9 terms), development (7 terms), signaling (6 terms), and proliferation/apoptosis (5 terms) (Additional file 12). Many of these GO terms were previously enriched (EASE score ≤ 0.1) with the higher stringency (≤0.5 fold) repressed genes lists and here reveal more robust (Benjamini ≤0.05) enrichments. Indeed, 5 terms, including “growth factor activity”, two transcriptional control terms, “signal transduction” and “negative regulation of apoptosis”, showed significant enrichment (Benjamini ≤0.05) in all cell variants (Additional file 12). This again highlights repression of growth factors as a core aspect of glucocorticoid-mediated repression. The strong enrichment of “positive regulation of transcription from RNA pol II promoter” and “transcriptional activator activity, RNA pol II core promoter binding” across repressed genes from all three cell variants, includes multiple inflammatory transcription factors (IRF1, JUN) and echoes the glucocorticoid-mediated suppression of transcription. However, despite such commonality, many stress response and proliferation/apoptosis terms were more robustly enriched in HBE cells, compared to A549 and BEAS-2B cells (Additional files 11 and 12).

### Budesonide-regulated gene expression in vivo

In a prior cross-over study, we reported glucocorticoid-modulated gene expression in the airways of 11 normal individuals, using microarray analysis of bronchial biopsies taken 6 h following a single inhalation of budesonide (1600 μg) or placebo [24]. Budesonide inhalation induced 63 (≥2 fold, *P* ≤ 0.05) and repressed 23 (≤0.5 fold, *P* ≤ 0.05) genes relative to placebo (Additional files 13 and 14). Combining these gene lists with those obtained for the three epithelial cell variants (Additional files 5 and 9) produced a total of 451 induced (≥2 fold; *P* ≤ 0.05) and 442 genes repressed (≤0.5 fold; *P* ≤ 0.05) in any cell variant or airway tissue (Additional files 13 and 14).

**Fig. 7:**
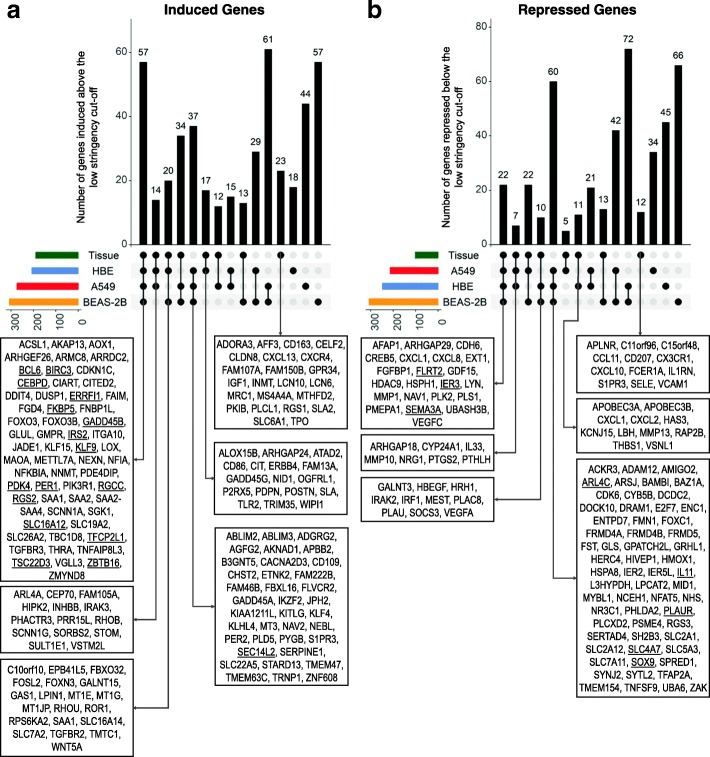
Comparison of budesonide-regulated genes in the three epithelial cell variants with those in airway tissue in vivo. Microarray analysis of A549 (*N* = 3), BEAS-2B (*N* = 4) and primary HBE (*N* = 4 donors) cells as well as airway tissue (*N* = 11 individuals) following budesonide exposure for 6 h identified 451 and 442 genes showing significant induction (≥ 2 fold, *P* ≤ 0.05) or repression (≤ 0.5 fold, *P* ≤ 0.05), respectively, compared to untreated control/placebo, in any of the cell variants or airway tissue. **a**. The 451 genes significantly induced (≥ 2 fold, *P* ≤ 0.05) by budesonide in any cell variant or airway tissue produced 15 groups showing overlaps as defined by the lower stringency, ≥ 1.25 fold cut-off, for each group. **b**. The 441 genes significantly repressed (≤ 0.5 fold, *P* ≤ 0.05) by budesonide in any cell variant or in airway tissue produce 15 groups showing overlaps, as defined by the lower stringency, ≤0.8 fold cut-off, for each group. Bars represent the number of genes within each group and below the genes in selected groups are listed. Genes within all groups are listed in Additional file 13 (for induced genes) and 13 (for repressed genes). Underlined genes represent genes that were in common between the cell variants using the stringent cut-off in Fig. 3 (for induced genes) and Fig. 5 (for repressed genes)

Comparing budesonide-induced genes (≥2 fold, *P* ≤ 0.05) in the airway epithelial cell variants and tissue revealed seven genes (ERRFI1, FKBP5, KLF9, PDK4, PER1, TFCP2L1, TSC22D3) that were induced in common. As above, a less stringent cut-off (≥1.25 fold) was then applied to the same pool of genes (Additional file 13). This identified 57 genes that were induced in common in vivo (in the airway tissue) and in primary HBE cells, plus the two cell line models (Fig. 7a). These genes include transcriptional regulators (CEBPD, CITED2, FOXO3, KLF9, KLF15, NFKBIA, PER1, TSC22D3, ZBTB16), proliferation/apoptosis-associated genes (AKAP13, BCL6, DUSP1, FGD4, FOXO3, GADD45B), and signaling genes (AKAP13, ARHGEF26, ERRFI1, FGD4, FNBP1L, IRS2, RGS2). Furthermore, the 17 in common genes induced ≥2 fold (*P* ≤ 0.05) by budesonide in the cell variants (Fig. 3a) were, with the exception of SEC14L2, all induced at least 1.25 fold in the airway tissue (underlined in Fig. 7a). Functionally, these 57 genes are a subset of those genes induced in common in the 3 cell variants and therefore carry a similar GO signature (Fig. 3b).

The above analysis identified 37 genes that were induced in common among the three cell variants, but which were not appreciably induced in airway tissue (Fig. 7a; Additional file 13). While this may be due to gene expression patterns gained during in vitro propagation of cells, such genes could represent epithelial-specific genes, which, due to dilution effects, are not captured in the heterogeneous cell population in the biopsy. Conversely, the 23 genes, including CD163, CXCR4, and PLCL1, induced in the airways tissue, but not the cell variants may represent non-epithelial effects or possibly responses lost in culture (Fig. 7a). Additional groups of interest includes those genes induced in common between airway tissue, primary HBE, that can be modelled in either A549 (14 genes including IRAK3 and SCNN1G) or BEAS-2B (20 genes that include GAS1 and WNT5A) cells. Notably, 17 genes (including ALOX15B, and TLR2) were induced by budesonide in HBE cells and airway tissue, but not in the cell lines (Fig. 7a). Such features are likely to be “real”, but are not readily modeled in A549 or BEAS-2B cells.

Comparing the budesonide-repressed genes using the stringent cut-off (fold ≤0.5, *P* ≤ 0.05) showed that none of the 23 genes repressed in airway tissue were common with the airway epithelial cell variants (Additional file 14). Applying the less stringent (fold ≤0.8) fold cut-off to the same pool of genes, showed 22 to be repressed in common in all three cell variants (Fig. 7b). However, this group included only 3 of the 8 genes showing repression in common in the cell variants using the stringent cut-off (Fig. 5a, underlined in Fig. 7b). Given that bronchial biopsies may contain only a small fraction of epithelial cells, dilution effects may explain the failure to detect repression that occurred only in epithelial cells. Nevertheless, these repressed in common genes are likely to represent key glucocorticoid-mediated effects and should be considered in functional assessments (Fig. 7b). Additionally, the low number of repressed genes from airway tissue resulted in larger groups of shared features between the cell variants compared with those involving tissue (Fig. 7b). As a consequence, only 11 genes were repressed in common between airway tissue and primary HBE cells, with 7 and 10 genes repressed in common between tissue, HBE cells and either A549, or BEAS-2B, respectively. An additional group of interest are the 12 genes, including CD207, CX3CR1, CXCL10, FCER1A, and VCAM1, that were uniquely repressed in airway tissue and which are likely to be of non-epithelial origin. In fact, the expression of genes, such as CXCL10, was enhanced in the presence of inflammatory stimuli in A549 and BEAS-2B cells, but was selectively maintained, or only slightly repressed, by glucocorticoids in these cells [40, 41]. Thus, the repression in the airway tissue is likely to be due to non-epithelial influences.

### Pathway analysis of budesonide-modulated genes

The above analyses suggest that even genes with low levels of differential expression, i.e. fold ≥1.25 or ≤ 0.8, are likely to represent genuine effects of budesonide and may collectively contribute to glucocorticoid function. In A549, BEAS-2B, HBE cells, and the airway tissue, these cut-offs (fold ≥1.25 or ≤ 0.8, *P* ≤ 0.05) identify 904, 1504, 757, and 553 induced genes and 1033, 1687, 923, and 341 repressed genes, respectively. These were then applied to the analysis of known pathways using: i, Ingenuity Pathway Analysis (IPA), which utilizes expression changes to produce an activation/inhibition score of the associated pathways, and; ii, simple enrichment of KEGG (Kyoto Encyclopedia of Genes and Genomes) pathways terms using DAVID.

Pathway analysis using IPA returned 55 pathways showing significant enrichment of budesonide regulated, induced or repressed, genes in any of the airway epithelial cell variants or airway tissue (Additional file 15). Various inflammatory pathways showed marked inhibition by budesonide in all cell variants and airway tissue, including “NF-κB signaling”, “TNFR1 and 2 signaling”, “B cell activating factor signaling”, “osteoarthritis pathway” and “toll-like receptor signaling”. Conversely, “Th1 Pathway” was activated in all 3 cell variants and airway tissue, suggesting that glucocorticoids may generally maintain, or even enhance, Th1 responses, while supressing Th2 activity. Similarly, “IL-2 Signaling pathway” showed activation by glucocorticoid-regulated genes in all the cell variants and airway tissue. While many of these results may be due to the presence of genes that contribute to multiple related pathways, they underpin roles for epithelial cells in shaping the immune response. Pathways that are likely to be involved in the metabolic and proliferative effects of glucocorticoids were also pronounced. For example, “PTEN signaling” was inhibited in common among the cell variants and airway tissue, whereas the term “type I diabetes mellitus signaling” was inhibited in cells, but not in airway tissue. Activated pathways that are likely related to metabolic effects of glucocorticoids include “IGF-1 signaling”, “PPAR signaling”, and “renin-angiotensin signaling” (Additional file 15). Notably, many of the enriched pathways in this analysis were likely to be activated in the airway tissue, while the activation/inhibition z-score for the epithelial cell variants suggested variable or often opposite effects (Additional file 15). This may reflect the influence of multiple mediators that are present in the airways, rather than just the influence of glucocorticoids, as would be the case in the cultured cells. Furthermore, the multiple cell types in the airway tissue may produce differential effects that differentially contribute to the overall response.

KEGG pathway analysis using DAVID revealed 40, 37, 32, and 34 pathways that were enriched (EASE score ≤ 0.1) with budesonide-induced genes (≥1.25 fold, *P* ≤ 0.05) in A549, BEAS-2B, HBE cells, and airway tissue, respectively (Additional file 16). Four pathways; “FoxO signaling pathway”, “PI3K-Akt signaling pathway”, “Mineral absorption”, and “Aldosterone-regulated sodium reabsorption”, were enriched (EASE score ≤ 0.1) in common in all three epithelial cell variants and airway tissue (Table 1). In fact, more robust enrichments (Benjamini ≤0.05) were observed for “FoxO signaling pathway” and “Aldosterone-regulated sodium reabsorption” in airway tissue, and for “PI3K-Akt signaling pathway” in A549 cells. Four additional pathways (“Signaling pathways regulating pluripotency of stem cells”, “Insulin signaling pathway”, “Toxoplasmosis” and “Pathways in cancer”) were enriched in common between HBE cells, airway tissue, and either A549 or BEAS-2B cells (Table 1).

**Table 1:**
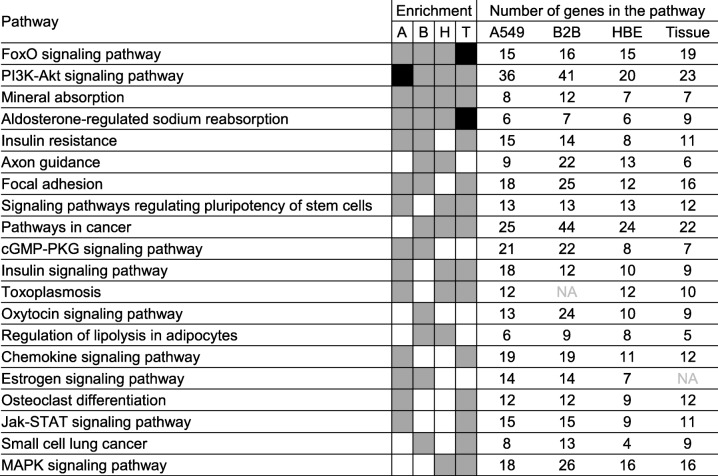
Top 20 pathways enriched by budesonide-induced genes in airway epithelial cell variants and tissue

Budesonide-induced genes ≥1.25 fold (*P* ≤ 0.05) in A549 (904 genes), BEAS-2B (1504 genes), HBE (757 genes), and airway tissue (553 genes) were subjected to KEGG pathway analysis using DAVID. KEGG pathways that were significantly enriched (EASE score ≤ 0.1) with each list and represented by at least 5 genes were obtained. The top 20 pathways, as defined by the product of the enrichment *P* values from all cells and tissue, are shown. Grey indicates significantly enriched pathways (EASE score ≤ 0.1). Black indicates pathways that are significantly enriched following family-wide false discovery rate correction (Benjamini ≤0.05). Pathways are sorted according to the product of *P* values in an ascending order. A = A549, B = BEAS-2B, H = HBE, and T = airway tissue (biopsy)

KEGG pathway analysis of budesonide-repressed genes (≤0.8 fold, *P* ≤ 0.05) showed enrichment for 25, 44, 37, and 17 pathways in A549, BEAS-2B, HBE cells, and airway tissue, respectively (Additional file 17). Two pathways; “TNF signaling pathway” and “Pathways in cancer” were common to all with TNF signaling pathway being most robustly enriched (Benjamini ≤0.05) in HBE cells and airway tissue, and “Pathways in cancer” showing Benjamini ≤0.05 in BEAS-2B cells (Table 2). “Cytokine-cytokine receptor interaction” was common between HBE cells and airway tissue, and “NF-kappa B signaling pathway” or “MAPK signaling pathway” were further in common with either A549 or BEAS-2B cells.

**Table 2:**
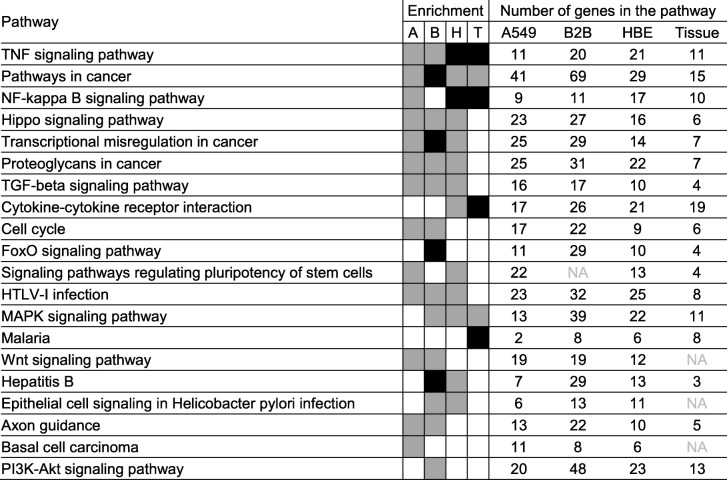
Top 20 pathways enriched by budesonide-repressed genes in airway epithelial cell variants and tissue

Budesonide-repressed genes ≤0.8 fold (*P* ≤ 0.05) in A549 (1033 genes), BEAS-2B (1687 genes), HBE (923 genes), and airway tissue (341 genes) were subjected to KEGG pathway analysis using DAVID. KEGG pathways that were significantly enriched (EASE score ≤ 0.1) with each list and represented by at least 5 genes were obtained. The top 20 pathways, as defined by the product of enrichment *P* values from all cells and tissue, are shown. Grey indicates significantly enriched pathways (EASE score ≤ 0.1). Black indicates pathways that are significantly enriched following family-wide false discovery rate correction (Benjamini ≤0.05). Pathways are sorted according to the product of *P* values in an ascending order. A = A549, B = BEAS-2B, H = HBE, and T = airway tissue

## Discussion

As airway epithelial cells are critical in asthma and mediate key responses to ICS [20, 21, 23], gene expression changes in response to maximally effective concentrations of the ICS, budesonide, were compared in two epithelial cell lines and primary HBE cells. Importantly, the effect of the ICS, budesonide, on global gene expression change was synonymous with that for dexamethasone in A549 cells and this suggests that current data will apply to glucocorticoids as a class. Initial kinetic analyses focused on BEAS-2B cells where budesonide induced 63 genes (≥2-fold, *P* ≤ 0.05) at 1 h, 95% of which were also among the 136 genes induced at 2 h. In contrast, only 15 genes showed repression (≤0.5-fold, *P* ≤ 0.05) at 1 h and < 60 genes were repressed at 2 h. Thus, although rapid onset glucocorticoid-dependent repression occurs, GR acts primarily as a transcriptional activator at these early times. However, by 6 h, the budesonide-induced and -repressed genes were apparent in larger and roughly equal numbers (240 and 236, respectively). At 18 h, the induced genes had plateaued at ~ 260 and the repressed genes increased to 347. This delay in repression relative to induction was previously reported in A549 cells and may simply reflect that mRNA loss takes time to manifest following reduced transcription [42]. However, mRNAs induced early, 1–2 h peak, revealed numerous, positive and negative, transcriptional regulators. These included CEBPB, FOXC2, KLF4, KLF6, KLF9 and PER1, and functional annotation clustering (not shown) confirmed multiple enriched GO terms for transcriptional regulation. Thus, GR rapidly induces expression of numerous transcriptional regulators that may mediate later-onset induction or repression of gene expression [43, 44], possibly via feed-forward loops involving GR [45].

Relating the above to inflammation (and repair) shows that while some mRNAs (IL6, IL11, and JUN) are highly repressed 1 h following budesonide, the repressive effects on most mRNAs, including transcription factors (FOSL1, ETS1), mediators (CCL2, NGF), and receptors (TNFRSF11B, IL7R), is more delayed. While such repressive effects may occur via GR transrepression and involve tethering or cis-acting nGREs, with co-repressor and HDAC recruitment [4–7], GR need not directly cause repression. GR-dependent transactivation of repressors of gene expression and/or signaling could suffice. For example, TSC22D3 represses NF-κB and AP-1 and is glucocorticoid-induced in multiple cell types, as well as in the human airways and in asthmatics taking ICS [24, 30, 46]. In BEAS-2B cells, TSC22D3 was the most highly budesonide-induced gene at both 1 and 2 h (Additional file 3). Similarly, glucocorticoid induction of NFKBIA, also known as IκBα, and TNFAIP3, or A20, may promote repression of NF-κB [15, 47]. Similarly, glucocorticoids induce the phosphatase, DUSP1, to reduce MAPK activity [48, 49], and ZFP36 [50], to destabilize and reduce translation of AUUUA-containing inflammatory mRNAs [15, 51]. Likewise, the up-regulation of IRAK3, a dominant negative inhibitor of signaling from MyD88, will reduce responses from core inflammatory receptors, including IL1 and toll-like receptors [52]. Such data are therefore consistent with the concept that glucocorticoids up-regulate multiple negative regulators of inflammatory pathways [53], including NF-κB and AP-1, as recently reported in LPS-treated macrophages [54]. However, the current analysis highlights the induction of various additional genes, including KLF9 [44], PER1 [55], and ZBTB16 [56], for which transcriptional repression properties are also apparent. Indeed, TSC22D3, PER1 and KLF9 were ≥ 2 fold induced in all three epithelial cell variants and using the lower stringency (≥1.25-fold cut-off) brings DUSP1, NFKBIA and ZBTB16 into this group of commonly induced repressors. This may explain the widely reported ability of protein synthesis inhibitors to block glucocorticoid-mediated repression of many genes [11, 14, 16, 57]. Not only are cytokines, chemokines and other inflammatory genes repressed in BEAS-2B cells, but GO analysis in all three epithelial cell variants reveal transcription and signaling, as well as specific terms for “cytokine activity” or “chemotaxis” to be targets of glucocorticoid repression. Many of these effects occur in vivo (see enrichment of GO terms including “cytokine activity”, “cytokine-receptor interaction” or “inflammatory response” with the budesonide-repressed genes in airway biopsy tissue post-budesonide inhalation) and this provides confidence that these are genuine effects of an ICS [24].

GO terms for the regulation of apoptosis and effects on cell proliferation were also enriched with the budesonide-induced genes. Thus, BCL6, a proto-oncogene from the same family as ZBTB16 [58], and BIRC3, also known as cellular inhibitor of apoptosis 2, are up-regulated in the three epithelial cell variants and in the airways in vivo. Clearly, there is also a relationship with repressive effects on the NF-κB pathway as this is generally anti-apoptotic [59]. For example, NFKBIA and TNFAIP3, appear under GO terms for apoptosis. Similarly, ZBTB16 reduces inflammatory gene expression, is implicated in cell cycle control and is pro-apoptotic [56, 60]. However, it remains unclear to what extent glucocorticoids promote epithelial cell apoptosis. High concentrations (> 3 μM) of dexamethasone or budesonide may certainly promote apoptosis in 1HAEo^−^ cells or primary HBE cells [61], but in A549 cells glucocorticoids were anti-apoptotic [62]. This discussion is complicated by the fact that many budesonide-repressed genes (including; BMP4, IER3, IL6, PLAUR, SOCS2/3 & SOX9) show enrichment of GO terms for apoptosis, in particular “negative regulation of apoptotic process”. This aspect of GR biology is highlighted by the “FOXO signaling pathway”, which is involved in cell cycle control and apoptosis [63], and was enriched in the budesonide-induced gene lists from all three epithelial cell variants and the airway tissue. Many such genes are also intertwined with positive and negative GO terms for cell proliferation, which are enriched in both the budesonide-induced and -repressed gene lists. For example, “cell proliferation” and “PI3K-Akt signaling pathway”, which contain growth factors, cytokine receptors as well as JAK-signaling, were enriched in the budesonide-induced gene lists and may, along with FOXO signaling, control cell survival and apoptosis [64]. Similar positive effects of ICS on cell signaling, proliferation and movement were indicated in airways biopsies taken 6 h post-budesonide inhalation [24]. Conversely, the GO term “growth factor activity” was significantly enriched in the repressed lists for each epithelial cell variant. Thus, the overall data indicate wide-ranging, but sometimes divergent effects of glucocorticoids on cell apoptosis, proliferation and cell movement. While glucocorticoids are anti-inflammatory, endogenous roles include healing and repair [65]. This not only includes reduced chemokine expression and loss of chemotaxis, but also reduced expression of many mediators that promote proliferation and activation, while promoting apoptosis of inflammatory and other cells. However, healing may involve migration to damaged regions prior to a cessation of migration and division to allow differentiation and final repair. Thus the many, apparently conflicting, GO terms may relate to normal physiological roles.

One area of cell-type-dependent regulation may occur in the coagulation and complement activation cascades. In the three airway epithelial cell variants, PLAUR, the urokinase plasminogen activator receptor, was strongly repressed by budesonide. PLAUR is necessary for cell-surface binding of urokinase plasminogen activator, PLAU, which is also repressed by budesonide in BEAS-2B and HBE cells, as well as in the airways tissue. Thus, reduced formation of plasmin, from plasminogen, is predicted at epithelial surfaces. While expression of PLAT, tissue plasminogen activator, is variably repressed (A549), activated (BEAS-2B) or not significantly affected (HBE cells, airways tissue) by budesonide, enhanced expression of SERPINE1, encoding plasminogen activator inhibitor 1 (PAI-1), will reduce the ability of PLAU and PLAT to activate plasmin. These findings are relevant as plasmin mediates fibrinolysis, which is reduced by glucocorticoids [66]. Indeed, inhaled budesonide was suggested to up-regulate coagulation and complement cascades in the airways [24]. In the context of the epithelium, fibrinolysis may be depressed. Furthermore, while clotting cascades are implicated in inflammation, fibrosis, and remodelling [67, 68], SERPINE1 is also implicated in pro-fibrotic remodeling [68]. Thus, while glucocorticoids can modulate such processes, details of activation/repression combined with timing and location may be critical to appreciate physiological roles. This may integrate with increased expression of genes involved in cytokine signaling, PI3K, Akt, FOXO, mTOR pathways and others (this study, [24]), and may collectively impact on wound repair, healing and/or alternatively on unwanted remodeling [69–71].

While the current analysis compared responses to budesonide in airway epithelial cells, commonality was surprisingly modest. Of the 187, 240, and 86 genes (total 410 individual genes) induced (≥2-fold, *P* ≤ 0.05) in A549, BEAS-2B or HBE cells, respectively, only 17 were common, of which 7 were also induced in the airway tissue. Despite this, heat maps showed that the direction of change was more conserved and could be captured by lowering the cut-off criteria to 1.25 fold within the 410 genes induced ≥2-fold (*P* ≤ 0.05) in any of the epithelial cell variants. This gave 93 genes induced in common, a majority of which (57 genes) were also up-regulated in vivo. Validation by qPCR confirmed this level of change in > 95% of these genes and in general showed the microarrays to under-report fold change. Functionally, while relevant responses can be driven by genes showing high fold change, the net effect of many genes, each showing smaller changes, on an outcome or pathway, may be significant. This further justifies use of the lower threshold in GO analyses. Indeed, observations made with the stringent expression criteria were confirmed and generally strengthened by the less stringent inclusion criteria. This supports the idea that functional effects of highly regulated genes can be reinforced by more modest changes in multiple other genes.

The identified gene expression commonalities provide a strong rationale for mechanistic and/or functional analyses of gene expression changes occurring in primary HBE cells, and/or in vivo, and which can be modelled in either, or both, of the two cell lines. However, the selection of the most appropriate model for mechanistic analysis may be critical. Both cell lines reveal commonalities with primary cells, and often the airway tissue, that may not be shared by the other. Conversely, analysis of changes occurring in the cell lines, but not in primary cells, or in vivo, may be more difficult to justify. This raises questions as to what should be considered as “real” expression change? Which of these effects may genuinely occur in cells in vivo, as opposed to merely being artefacts of culture, or cell transformation. This is difficult to address. A549 cells are adenocarcinoma cells derived from type II alveolar cells [72, 73], whereas BEAS-2B cells are SV40 transformed bronchial airway epithelial cells [74]. Both the origin of the parent cells and reasons for immortality will impact on gene expression. Likewise, culture media composition is different for each variant. However, it is also true that each cell line represents a different genetic background, as does each HBE donor. It is therefore possible that genetic, and very likely epi-genetic, differences between individuals could contribute to the diversity of responses. Analysis of these issues is warranted.

## Conclusions

In conclusion, the current analysis characterizes gene expression patterns following budesonide treatment of airway epithelial cell lines and primary HBE cells. Repressive effects of glucocorticoids are confirmed on inflammatory pathways and genes, and repression of growth factors is highlighted. The early response to glucocorticoid primarily shows transcriptional activation, in particular, of positive and negative regulators of transcription, gene expression and signaling. This reveals how early GR transactivation may drive more numerous changes, both repression and activation, occurring at longer times post-glucocorticoid exposure. While many genes regulated in common are identified, considerable differences in the profiles between each cell line and HBE cells are exposed. This poses an issue for the interrogation of glucocorticoid-regulated function. Understanding this diversity will be essential to promote analysis of gene expression changes in the physiological and therapeutic effects of glucocorticoids.

## Additional files

Additional file 1: Oligonucleotides used for qPCR. Forward and reverse primer sequences (5′- 3′) are shown. For genes with more than one splice variant, primers were designed to detect all variants. All primers were designed using Primer BLAST (NCBI) and were synthesized by the DNA synthesis lab at the University of Calgary. (DOCX 19 kb)

Additional file 2: Concentration-dependent induction of gene expression by budesonide in primary human bronchial epithelial (HBE) cells. Primary HBE cells were either not treated or treated with the indicated concentrations of budesonide. After 6 h, cells were harvested for RNA and qPCR was performed for the indicated genes and GAPDH. Data (*N* = 3 individuals), normalized to GAPDH, were expressed as fold relative to untreated control and are plotted as mean ± SE. (PDF 21 kb)

Additional file 3: Budesonide-modulated genes at different times in BEAS-2B cells. The 820 genes showing significant induction (fold ≥2, *P* ≤ 0.05), or repression (fold ≤0.5, *P* ≤ 0.05), by budesonide (300 nM), compared to control, in BEAS-2B cells at 1, 2, 6 or 18 h are listed along with the fold change and *P* values at each time. The time at which each gene shows the greatest absolute log_2_ fold change is indicated. Genes are sorted alphabetically according to their official gene symbol. (XLSX 92 kb)

Additional file 4: Genes significantly modulated by budesonide or dexamethasone in A549 cells. The 330 genes showing significant induction (fold ≥2, *P* ≤ 0.05), or repression (fold ≤0.5, *P* ≤ 0.05), by budesonide (300 nM) or dexamethasone (1 μM), compared to control at 6 h, are listed along with the fold change and *P* value for each glucocorticoid. Genes are sorted alphabetically according to their official gene symbol. (XLSX 30 kb)

Additional file 5: Budesonide-induced genes in A549, BEAS-2B or HBE cells. The 410 genes that were significantly induced ≥2 fold (*P* ≤ 0.05) by budesonide at 6 h in A549 (A), BEAS-2B (B), or HBE (H) cells are listed. Last two columns (I and J), indicate the gene groups for each gene based on the induction thresholds, ≥2 fold (*P* ≤ 0.05) or ≥ 1.25, as shown in Fig. 3. The 52 genes that were selected for qPCR validation are colored red. Genes are sorted alphabetically according to their official gene symbol. (XLSX 45 kb)

Additional file 6: Validation of budesonide-induced genes grouped by fold ≥1.25 cut-off. Figure 3b defines 7 groups, representing overlaps and unique genes (≥ 1.25 fold cut-off) within the 410 genes that were induced ≥2 fold (*P* ≤ 0.05) by budesonide in the three cell variants. Overall gene expression data for each of these 7 groups was summarized by: **a**, zero-mean log_2_ intensity, and; **b**, the log_2_ fold change when compared to untreated control. Data are color-coded (Red = A549, yellow/orange = BEAS-2B, Blue = HBE). In panel **a**, open bars represent untreated and solid bars are budesonide-treated cells. The box defines the upper and lower quartiles, and the line inside represents the median. Whiskers represent the 5th and 95th percentiles. **c.** Following either no treatment or budesonide (300 nM for A549 and BEAS-2B, 100 nM for HBE) for 6 h, real-time PCR was performed for the indicated genes and GAPDH in A549 (*N =* 6), BEAS-2B (*N =* 6) and HBE (*N =* 6 donors). Data, representing log_2_ fold change for each gene/GAPDH, are presented as scatter plots (from different experimental replicates) along with their means ± SE. Following qPCR, 19 genes had their grouping changed. The revised designations are indicated in green (15 upgraded genes) or red (4 downgraded genes), where A = A549, B = BEAS-2B and H = HBE. **d**. Correlation between the log_2_ fold change produced by budesonide treatment as obtained by microarray analysis and that acquired by real-time qPCR analysis in each cell variant. The dashed line represents the line of unity and the solid line represents the best fit line. (PDF 1730 kb)

Additional file 7: GO terms associated with budesonide-induced genes (≥2 fold, *P* ≤ 0.05) in A549, BEAS-2B, and HBE cells. Budesonide-induced genes ≥2 fold (*P* ≤ 0.05) in A549 (187 genes), BEAS-2B (240 genes), and HBE (86 genes) were subjected to GO analysis using DAVID. Biological processes (BP) and molecular function (MF) terms that were significantly enriched (EASE score ≤ 0.1) with each list and represented by at least 5 genes are listed. GO terms that are significantly enriched following family-wide false discovery rate correction (Benjamini ≤0.05) are colored red. (XLSX 26 kb)

Additional file 8: GO terms associated with budesonide-induced genes in A549, BEAS-2B, and HBE cells using lower stringency cut-off. Budesonide-induced genes, as defined in Fig. 3b, in A549 (268 genes), BEAS-2B (300 genes), and HBE (196 genes) were used for DAVID GO analysis. Biological processes and molecular function terms that were significantly enriched (EASE score ≤ 0.1) with each list and represented by at least 5 genes were listed. The terms that are significantly enriched following family-wide false discovery rate correction (Benjamini ≤0.05) are colored red. (XLSX 38 kb)

Additional file 9: Budesonide-repressed genes in A549, BEAS-2B or HBE cells. The 425 genes that were significantly repressed ≤0.5 fold (*P* ≤ 0.05) by budesonide at 6 h in A549 (A), BEAS-2B (B), or HBE (H) cells are listed. The last two columns (I and J), indicate the gene groups for each gene based on the induction thresholds, ≤0.5 fold (*P* ≤ 0.05) or ≤ 0.8, as shown in Fig. 5. Genes are sorted alphabetically according to their official gene symbol. (XLSX 49 kb)

Additional file 10: GO terms associated with budesonide-repressed genes (≤0.5 fold, *P* ≤ 0.05) in A549, BEAS-2B, and HBE cells. Budesonide-repressed genes ≤0.5 fold (*P* ≤ 0.05) in A549 (108 genes), BEAS-2B (236 genes), and HBE (146 genes) were subjected to GO analysis using DAVID. Biological processes (BP) and molecular function (MF) terms that were significantly enriched (EASE score ≤ 0.1) with each list and represented by at least 5 genes are listed. The terms that were significantly enriched following family-wide false discovery rate correction (Benjamini ≤0.05) are colored red. (XLSX 32 kb)

Additional file 11: GO terms associated with budesonide-repressed genes in A549, BEAS-2B, and HBE cells using the lower stringency cut-off**.** Budesonide-repressed genes, as defined in Fig. 5c, in A549 (211 genes), BEAS-2B (307 genes), and HBE (243 genes) were subjected GO analysis using DAVID. Biological processes (BP) and molecular function (MF) terms that were significantly enriched (EASE score ≤ 0.1) with each list and represented by at least 5 genes were listed. The terms that are significantly enriched following family-wide false discovery rate correction (Benjamini ≤0.05) are colored red. (XLSX 46 kb)

Additional file 12: Commonalities between GO terms enriched with budesonide-repressed (≤0.5 fold, *P* ≤ 0.05) genes in A549, BEAS-2B and HBE cells using the lower (≤0.8 fold) stringency cut-off between cell variants. The 425 budesonide-repressed genes, as defined in Fig. 5c (Additional file 9), were segregated between variants using the lower (≤0.8 fold) stringency cut-off. The 211 (A549), 307 (BEAS-2B) and 243 (HBE) genes for each cell variant were subjected to GO analysis using DAVID. Biological process and molecular function terms showing significant enrichment (EASE score ≤ 0.1) with each list, and represented by at least 5 genes, were obtained (Additional file 11). The number of GO terms enriched in each cell variant is indicated and the Venn diagram illustrates overlap in terms between the cell variants. To the right are the 41 GO terms that were enriched in common for all cell variants. Functional categorization is indicated. Significance, following correction for family-wide false discovery, in A549 (A), BEAS-2B (B), and HBE (H) cells is indicated where: Benjamini ≤0.05 (*), Benjamini ≤0.01 (**), or Benjamini ≤0.001 (***). (PDF 1470 kb)

Additional file 13: Budesonide-induced genes in airway epithelial cell variants and tissue. The 451 genes that were significantly induced ≥2 fold (*P* ≤ 0.05) by budesonide in A549 (A), BEAS-2B (B), HBE (H), or airway tissue biopsies (T) are listed. The last two columns (K and L), indicate the gene groups for each gene based on the induction thresholds, ≥2 fold (*P* ≤ 0.05) or ≥ 1.25. Genes are sorted alphabetically according to their official gene symbol. (XLSX 55 kb)

Additional file 14: Budesonide-repressed genes in airway epithelial cell variants and tissue. The 442 genes that were significantly repressed ≤0.5 fold (*P* ≤ 0.05) by budesonide in A549 (A), BEAS-2B (B), HBE (H), or airway tissue biopsies (T) are listed. The last two columns (K and L), indicate the gene groups for each gene based on the induction thresholds, ≤0.5 fold (*P* ≤ 0.05) or ≤ 0.8. Genes are sorted alphabetically according to their official gene symbol. (XLSX 57 kb)

Additional file 15: Pathways enriched in budesonide-regulated genes in airway epithelial cell variants and tissue were identified by Ingenuity Pathway Analysis. Budesonide-regulated genes ≥1.25 fold, or ≤ 0.8 fold (*P* ≤ 0.05), in A549 (1973 genes), BEAS-2B (3191 genes), HBE (1980 genes), and airway tissue (894 genes) were subjected to pathway analysis using Ingenuity Pathway Analysis (IPA). Pathways that were significantly enriched (−log *P* value ≥1.3; i.e. *P* ≤ 0.05) with any of the gene lists are shown. Enrichment (−log *P* value) is shown in purple and z-score is shown in red for positive values (activation) or in blue for negative values (inhibition). (XLSX 54 kb)

Additional file 16: KEGG pathways enriched in budesonide-induced genes in airway epithelial cell variants and tissue. Budesonide-induced genes ≥1.25 fold (*P* ≤ 0.05) in A549 (904 genes), BEAS-2B (1504 genes), HBE (757 genes), and airway tissue (553 genes) were subjected to KEGG pathway analysis using DAVID. KEGG pathways that were significantly enriched (EASE score ≤ 0.1) with each list and represented by at least 5 genes are listed. The terms that were significantly enriched following family-wide false discovery rate correction (Benjamini ≤0.05) are colored red. (XLSX 30 kb)

Additional file 17: KEGG pathways enriched in budesonide-repressed genes in airway epithelial cell variants and tissue. Budesonide-repressed genes ≤0.8 fold (*P* ≤ 0.05) in A549 (1033 genes), BEAS-2B (1687 genes), HBE (923 genes), and airway tissue (341 genes) were subjected to KEGG pathway analysis using DAVID. KEGG pathways that were significantly enriched (EASE score ≤ 0.1) with each list and represented by at least 5 genes are listed. The terms that were significantly enriched following family-wide false discovery rate correction (Benjamini ≤0.05) are colored red. (XLSX 30 kb)

## Abbreviations

All genes, unless otherwise indicated, are referred according the official gene symbols, as supplied by The National Center for Biotechnology Information (https://www.ncbi.nlm.nih.gov/). Other abbreviations are: C_T_: Threshold cycle
DAVID: Database for Annotation, Visualization, and Integrated Discovery
GO: Gene ontology
GR: Glucocorticoid receptor
GRE: Glucocorticoid response element
HBE: Human bronchial epithelial
ICS: Inhaled corticosteroid(s)
IPA: Ingenuity Pathway Analysis
KEGG: Kyoto Encyclopedia of Genes and Genomes
qPCR: quantitative polymerase chain reaction
