# Supplementary material for: Glucocorticoid-driven transcriptomes in human airway epithelial cells: commonalities, differences and functional insight from cell lines and primary cells
Source: BMC Med Genomics. 2019 Jan 31;12:29. doi: 10.1186/s12920-018-0467-2 (PMC6357449; doi:10.1186/s12920-018-0467-2)
Supplement: Supplementary file 1 — Oligonucleotides used for qPCR. Forward and reverse primer sequences (5′- 3′) are shown. For genes with more than one splice variant, primers were designed to detect all variants. All primers were designed using Primer BLAST (NCBI) and were synthesized by the DNA synthesis lab at the University of Calgary. (DOCX 19 kb) [file 12920_2018_467_MOESM1_ESM.docx]

| **Gene** | **Forward Primer (5ʹ-3ʹ)** | **Reverse Primer (5ʹ-3ʹ)** |
| --- | --- | --- |
| **ALOX5AP** | ACCGGAACACTTGCCTTT | CAAGTACATCAGTCCAGCAAAC |
| **BIRC3** | CCGTCAAGTTCAAGCCAGTTACCC | AGCCCATTTCCACGGCAGCA |
| **CD200** | GGACTGTGACCGACTTTAAGCAA | AGCAATAGCGGAACTGAAAAC |
| **CD86** | TTTGTGATGGCCTTCCTGCTC | GCTCACTCAGGCTTTGGTTTTG |
| **CDKN1C** | CGGCGATCAAGAAGCTGTC | GGCTCTAAATTGGCTCACCG |
| **CEBPB** | TCCAAACCAACCGCACAT | AGAGGGAGAAGCAGAGAGTTTA |
| **CEBPD** | GGAGATGCAGCAGAAGTTGGT | CGCGCTGGTGCAGCTT |
| **CNR1** | ATTTCGTTCTAGCGGACAACCA | TGACTGAGAAAGTGACCCACAG |
| **CRISPLD2** | CAAACCTTCCAGCTCATTCATG | GGTCGTGTAGCAGTCCAAATCC |
| **DOCK4** | GCTGTTTTCCCTTGCCACTG | ACCCTTTGCTCTCTTGCGAA |
| **DUSP1** | GCTCAGCCTTCCCCTGAGTA | GATACGCACTGCCCAGGTACA |
| **EIF2AK3** | GTCCCCCTGTGTGGTAACTG | TTACCCGCCAGGGACAAAAA |
| **FAM105A** | ACAGGCTCGAATGTGTTTGGA | GGGTGTTGCACATACTTCCTC |
| **FKBP5** | CAGCTGCTCATGAACGAGTTTG | GCTTTATTGGCCTCTTCCTTGG |
| **FOXO1** | CAAGAGCGTGCCCTACTTCA | GCACACGAATGAACTTGCTGT |
| **FOXO3** | CAAGGATAAGGGCGACAGCA | GGACCCGCATGAATCGACTA |
| **GAPDH** | TTCACCACCATGGAGAAGGC | AGGAGGCATTGCTGATGATCT |
| **GADD45A** | AGAAGACCGAAAGGATGGATAAG | AGGCACAACACCACGTTAT |
| **HIPK2** | GAGAATCACTGCACGGGGAA | GACGAGTGGTGACTGGTGTT |
| **IFIT1** | CCAACAGTGTAGTAGCCTCAAA | GGTGCGTCCTTAGAAGAAAGAG |
| **IRAK3** | ACCATGCTCGGTCATCTGTG | ATGTTCTAGGTGGGACCGGA |
| **ITGA10** | TCACTCACCTGTTCTTGCCC | GGAATAGGCGTGGGTGATGT |
| **JAG1** | AACAGATTCCAGTGTCTGTGTC | GCAGGGATTAGGCTCACAATAA |
| **KLF4** | TCGCCTTGCTGATTGTCTATT | AATTGGCCGAGATCCTTCTTC |
| **KLF6** | ACGAGACCGGCTACTTCT | CAGATCTTCCTGGCTGTCAAA |
| **KLF9** | CCTCCCATCTCAAAGCCCATT | TCGTCTGAGCGGGAGAACTT |
| **KLF15** | GCAGTGCATGTGCTTGAGTT | TTTGTCTGGGAAACCGGAGG |
| **LIFR** | ACATCATCAGCGTAGTGGCT | GGTCGTAATGCCAGGTGAGG |
| **MAOA** | TAGAAGCTCGGGACAGGGTT | TGGGTTGGTCCCACATAAGC |
| **MAP3K8** | ACTGTGGAGGATTTGCTTGCT | ACGTCCATTTTGGGGAGTGA |
| **MYOT** | ACTCTACCAAAGCCAGGAGCA | GCCTGGAATTACGGAAGGTTGT |
| **NCOA3** | GACAAACACCCCCAAGCAAC | CACCACCAGCAGTAGGGTTT |
| **NFKBIA** | TGGTGTCCTTGGGTGCTGAT | GGCAGTCCGGCCATTACA |
| **NNMT** | AAGGGAACAGAGTCAAGGGTC | GTCACATCACACTTCAGCACC |
| **PDE4DIP** | AGGGGATGACACCGAAGATAC | AAAGATGGGTTCTGGGTCTCC |
| **PDE6A** | TTTGATGTGTGGCCGGTTCT | AGGAGGTGGATTCGGGATGA |
| **PDK4** | GCGACAAGAATTGCCTGTGAG | TCCACCAAATCCATCAGGCTC |
| **PER1** | CGTCACCAGTCAGTGTAGC | CCCACTGGACGGTAGGC |
| **PHACTR3** | TTCTGAACTCAAAGACGACTTGCT | TGCATTTCCGTGGCAGTGT |
| **PTGER2** | ATGACCATCACCTTCGCCGT | AAGAGCTTGGAGGTCCCATTTT |
| **PTGER4** | TCTTACTCATTGCCACCTCCC | AAGCAATTCGGATGGCCTGC |
| **PTGS2** | GCTGGGCCATGGGGTGGACT | CCTGCCCCACAGCAAACCGT |
| **RGS2** | CCTCAAAAGCAAGGAAAATATATACTGA | AGTTGTAAAGCAGCCACTTGTAGCT |
| **SCNN1G** | CTTGGACCCTTTGGAACCGA | CATGGCGAGGATGGGACTTT |
| **SERPINE1** | CAGGCTGACTTCACGAGTCTT | ATGCGGGCTGAGACTATGAC |
| **SLC45A1** | TGCTGGGGCATGTGTATCTA | AGAGCAGCGAGTAAGGCAAG |
| **TFCP2L1** | GAGCTGGAGCAGAATCGAGTG | GATGCTGTACAGGTTGGCGAT |
| **TGFBR2** | TCTGGTGCTCTGGGAAATGAC | TCGCCCTCGATCTCTCAACA |
| **TLR2** | GCTGCTCGGCGTTCTCTC | AAGCAGTGAAAGAGCAATGGG |
| **TNFAIP3** | AGGCGCTGTTCAGCACGCTC | CGGGCCATGGGTGTGTCTGT |
| **TREM2** | ATGATGCGGGTCTCTACCA | ATCCTCGAAGCTCTCAGACTC |
| **TSC22D3** | GGCCATAGACAACAAGATCG | ACTTACACCGCAGAACCACCA |
| **ZBTB16** | GGGTCGAGCTTCCTGATAAC | TTCTCAGCCGCAAACTATCC |
