# Supplementary figures and images for: Glucocorticoid-driven transcriptomes in human airway epithelial cells: commonalities, differences and functional insight from cell lines and primary cells
Source: BMC Med Genomics. 2019 Jan 31;12:29. doi: 10.1186/s12920-018-0467-2 (PMC6357449; doi:10.1186/s12920-018-0467-2)

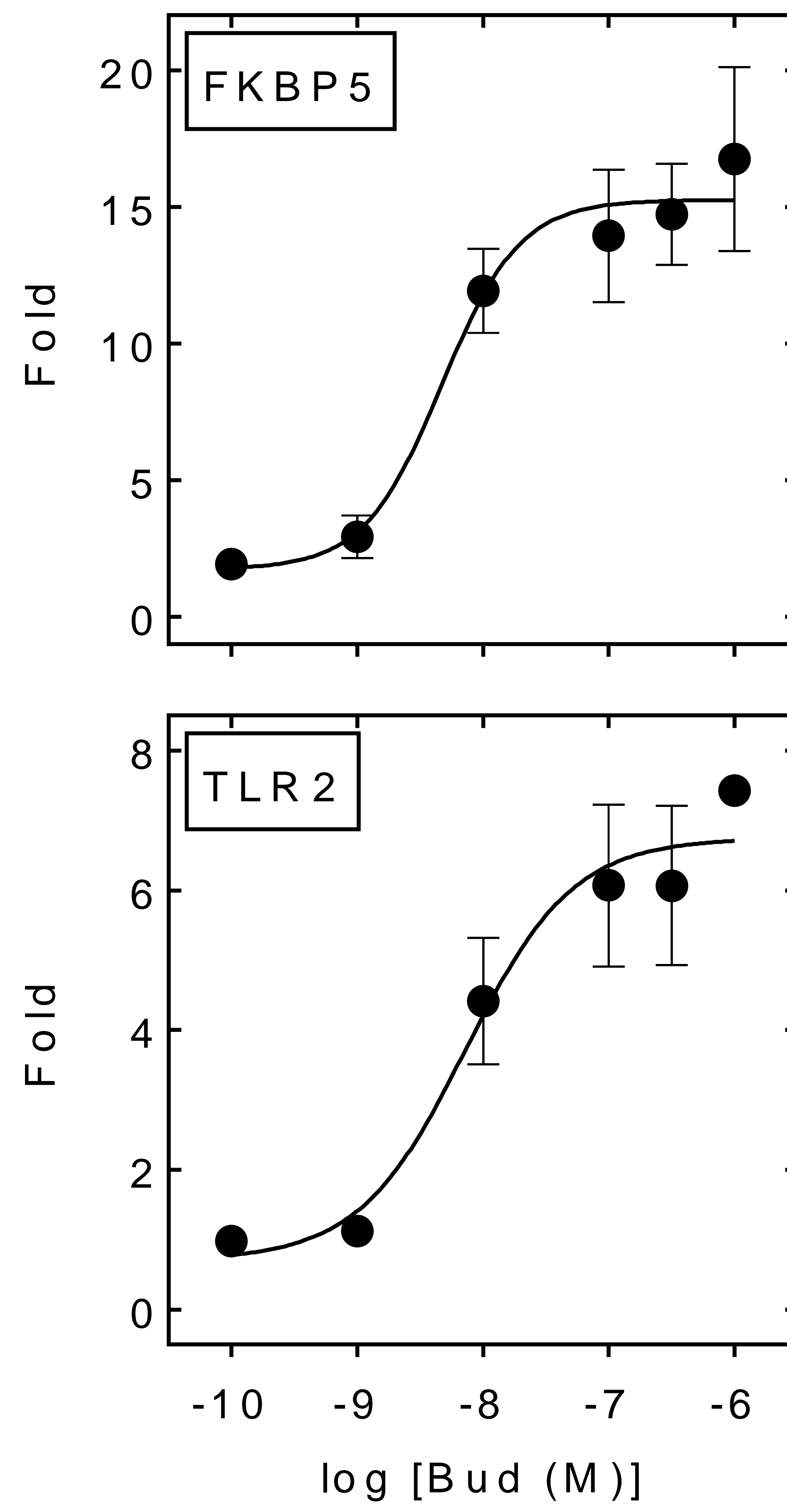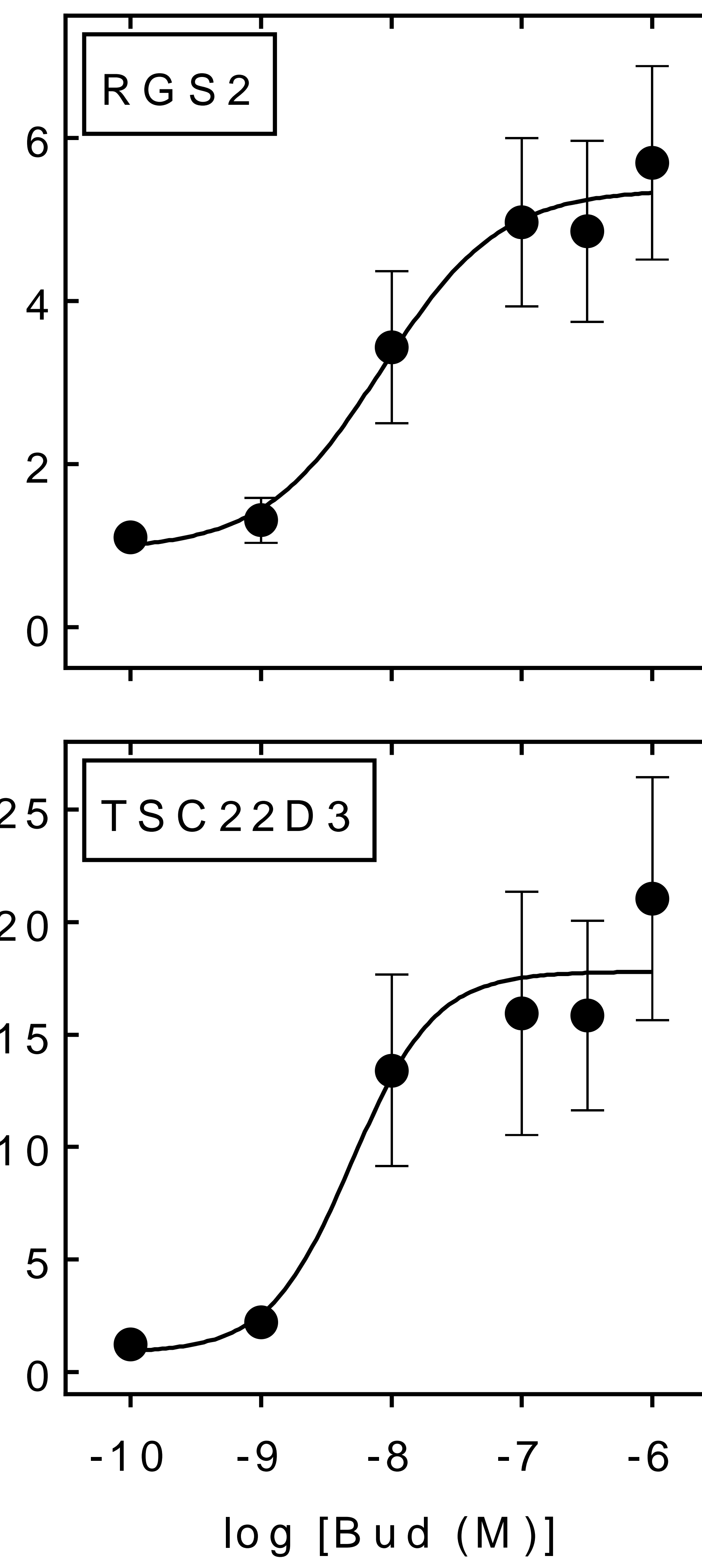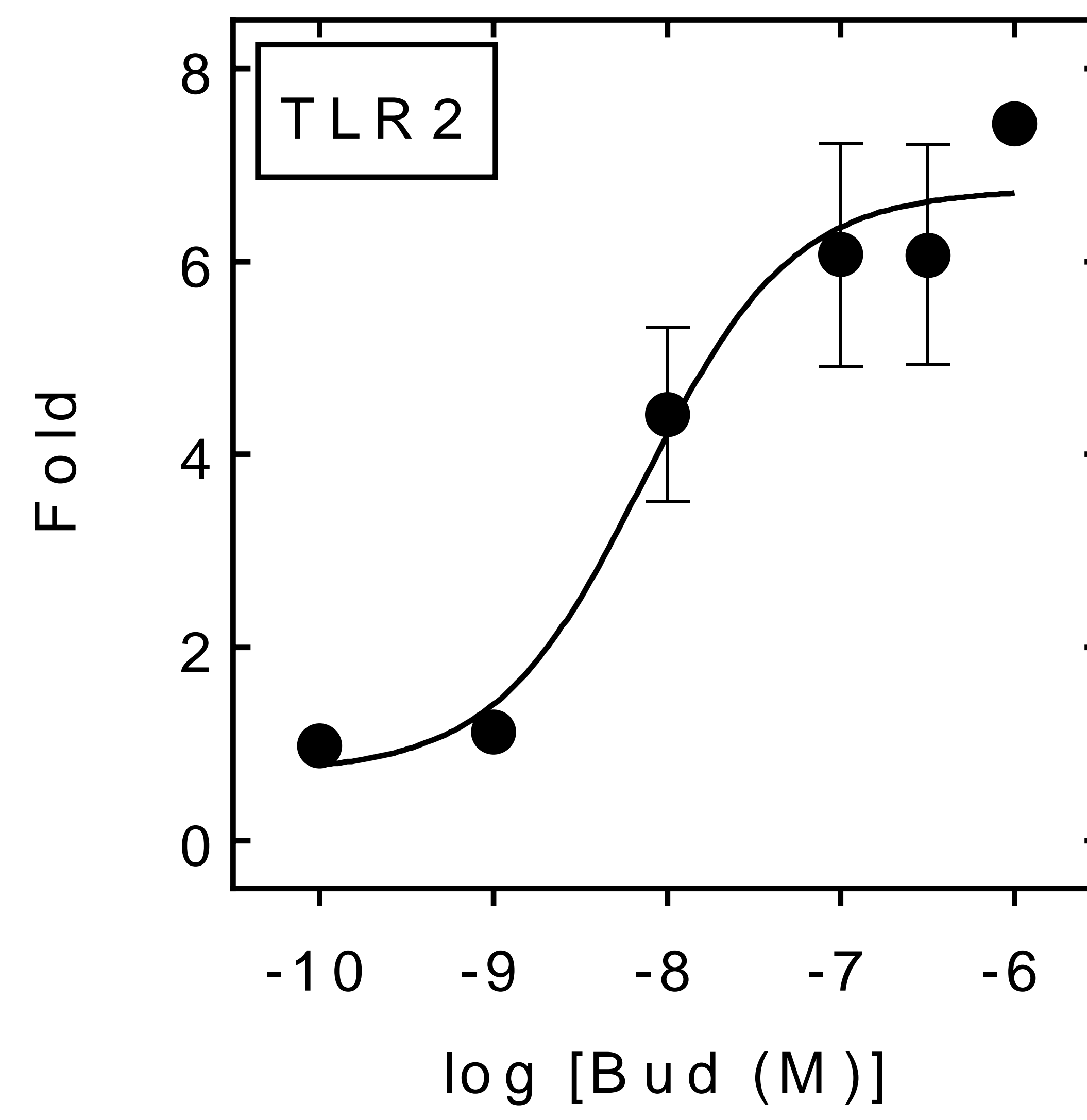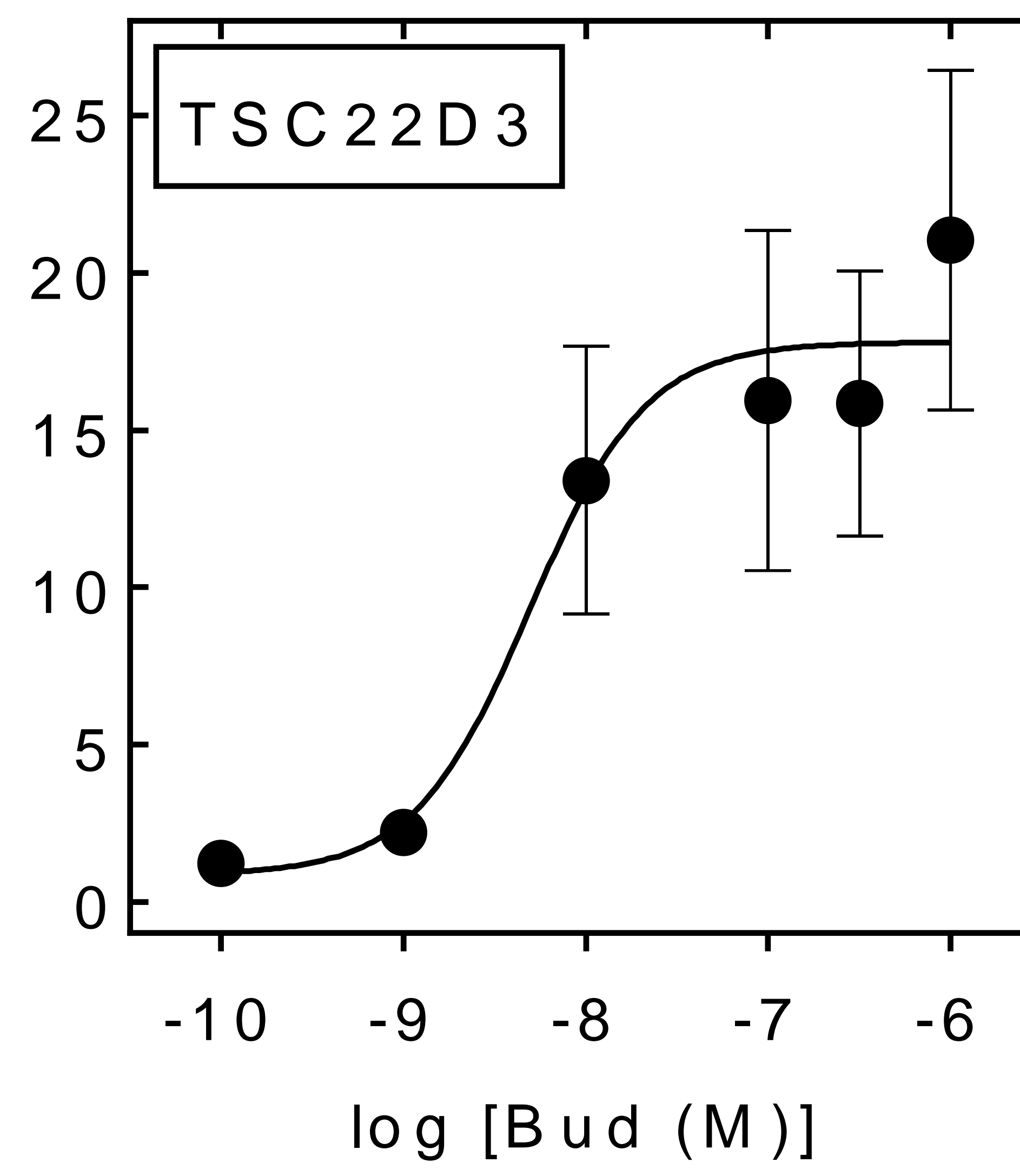

Supplement: Supplementary file 2 — Concentration-dependent induction of gene expression by budesonide in primary human bronchial epithelial (HBE) cells. Primary HBE cells were either not treated or treated with the indicated concentrations of budesonide. After 6 h, cells were harvested for RNA and qPCR was performed for the indicated genes and GAPDH. Data (N = 3 individuals), normalized to GAPDH, were expressed as fold relative to untreated control and are plotted as mean ± SE. (PDF 21 kb) [file 12920_2018_467_MOESM2_ESM.pdf]

## Microarray Summary

## qPCR Validation

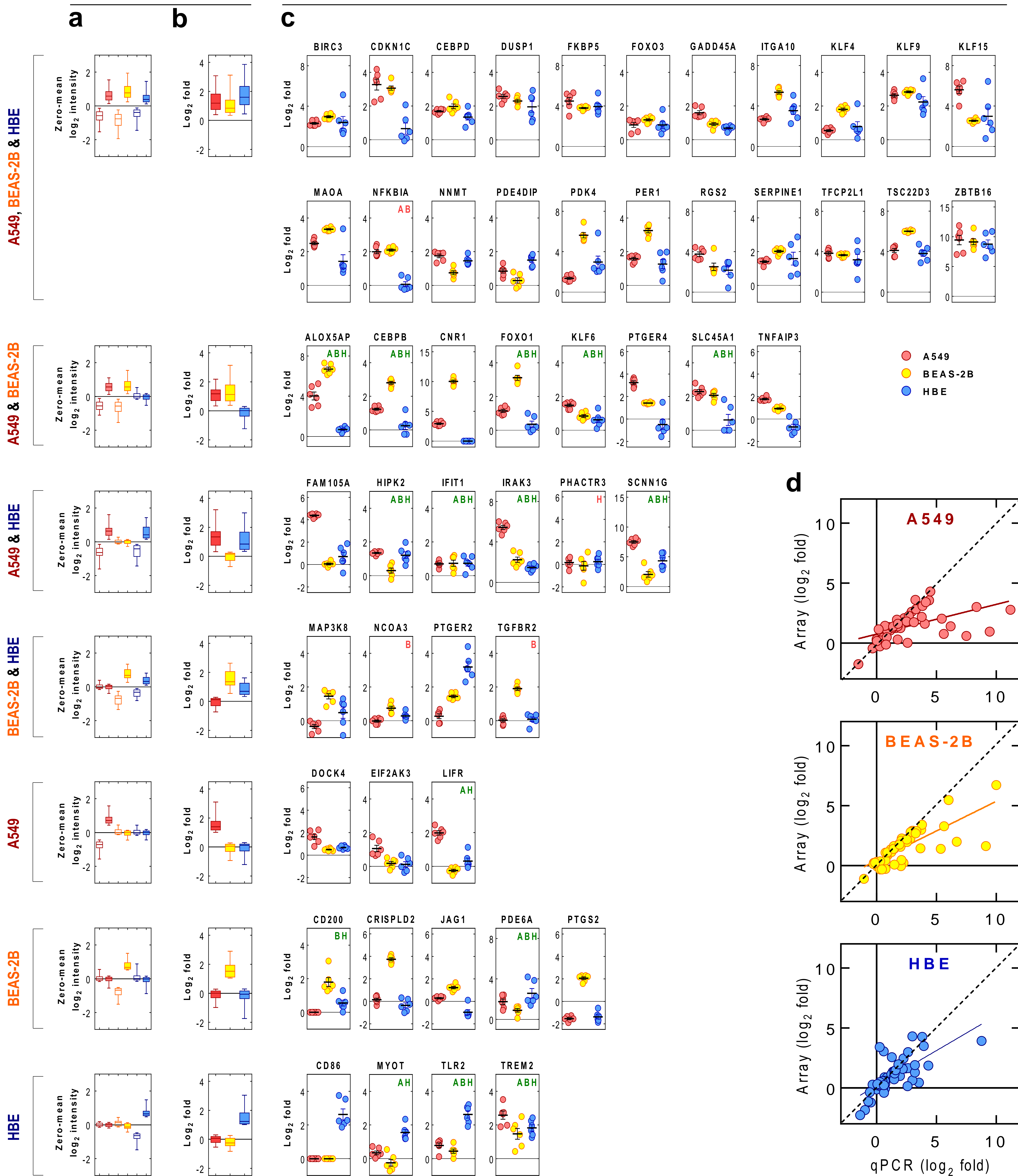

Supplement: Supplementary file 6 — Validation of budesonide-induced genes grouped by fold ≥1.25 cut-off. Figure 3b defines 7 groups, representing overlaps and unique genes (≥ 1.25 fold cut-off) within the 410 genes that were induced ≥2 fold (P ≤ 0.05) by budesonide in the three cell variants. Overall gene expression data for each of these 7 groups was summarized by: a, zero-mean log2 intensity, and; b, the log2 fold change when compared to untreated control. Data are color-coded (Red = A549, yellow/orange = BEAS-2B, Blue = HBE). In panel a, open bars represent untreated and solid bars are budesonide-treated cells. The box defines the upper and lower quartiles, and the line inside represents the median. Whiskers represent the 5th and 95th percentiles. c. Following either no treatment or budesonide (300 nM for A549 and BEAS-2B, 100 nM for HBE) for 6 h, real-time PCR was performed for the indicated genes and GAPDH in A549 (N = 6), BEAS-2B (N = 6) and HBE (N = 6 donors). Data, representing log2 fold change for each gene/GAPDH, are presented as scatter plots (from different experimental replicates) along with their means ± SE. Following qPCR, 19 genes had their grouping changed. The revised designations are indicated in green (15 upgraded genes) or red (4 downgraded genes), where A = A549, B = BEAS-2B and H = HBE. d. Correlation between the log2 fold change produced by budesonide treatment as obtained by microarray analysis and that acquired by real-time qPCR analysis in each cell variant. The dashed line represents the line of unity and the solid line represents the best fit line. (PDF 1730 kb) [file 12920_2018_467_MOESM6_ESM.pdf]
