# Supplementary material for: Glucocorticoid-driven transcriptomes in human airway epithelial cells: commonalities, differences and functional insight from cell lines and primary cells
Source: BMC Med Genomics. 2019 Jan 31;12:29. doi: 10.1186/s12920-018-0467-2 (PMC6357449; doi:10.1186/s12920-018-0467-2)
Supplement: Supplementary file 12 — Commonalities between GO terms enriched with budesonide-repressed (≤0.5 fold, P ≤ 0.05) genes in A549, BEAS-2B and HBE cells using the lower (≤0.8 fold) stringency cut-off between cell variants. The 425 budesonide-repressed genes, as defined in Fig. 5c (Additional file 9), were segregated between variants using the lower (≤0.8 fold) stringency cut-off. The 211 (A549), 307 (BEAS-2B) and 243 (HBE) genes for each cell variant were subjected to GO analysis using DAVID. Biological process and molecular function terms showing significant enrichment (EASE score ≤ 0.1) with each list, and represented by at least 5 genes, were obtained (Additional file 11). The number of GO terms enriched in each cell variant is indicated and the Venn diagram illustrates overlap in terms between the cell variants. To the right are the 41 GO terms that were enriched in common for all cell variants. Functional categorization is indicated. Significance, following correction for family-wide false discovery, in A549 (A), BEAS-2B (B), and HBE (H) cells is indicated where: Benjamini ≤0.05 (*), Benjamini ≤0.01 (**), or Benjamini ≤0.001 (***). (PDF 1470 kb) [file 12920_2018_467_MOESM12_ESM.pdf]

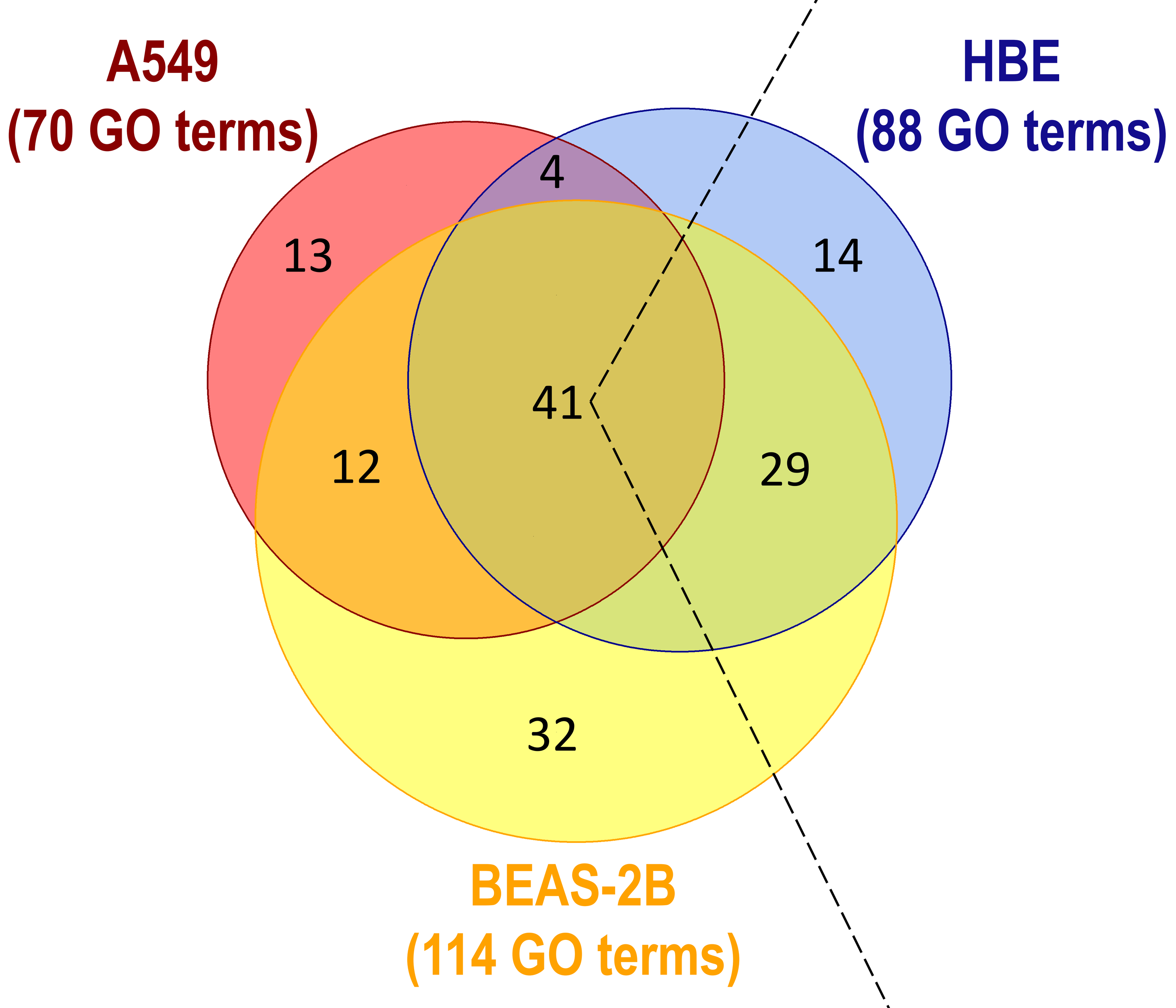

GO term	Benjamini		
	A	B	H
**positive regulation of transcription from RNA pol II promoter**	**	***	***
**transcriptional activator activity, RNA pol II core promoter binding**	***	**	**
positive regulation of transcription, DNA-templated		*	***
transcription from RNA pol II promoter		*	*
negative regulation of transcription from RNA pol II promoter		**	
negative regulation of transcription, DNA-templated			
RNA pol II core promoter proximal region sequence-specific DNA binding			
sequence-specific DNA binding			
transcription factor activity, sequence-specific DNA binding			
transcription factor binding			
transcription regulatory region DNA binding			
positive regulation of cell migration		***	*
chemorepellent activity	*		**
inflammatory response			***
response to hypoxia			*
cytokine activity			***
heparin binding		**	
chemotaxis			
immune response			
blood coagulation			
**growth factor activity**	*	**	***
angiogenesis		**	***
positive regulation of angiogenesis		*	**
organ morphogenesis	*		
axon guidance			
cell differentiation			
skeletal system development			
**signal transduction**	**	**	*
cell-cell signaling			**
positive regulation of ERK1 and ERK2 cascade		*	
positive regulation of GTPase activity		*	
intracellular signal transduction			
vascular endothelial growth factor receptor signaling pathway			
**negative regulation of apoptotic process**	*	*	*
negative regulation of cell proliferation		*	***
positive regulation of cell proliferation			***
apoptotic process			*
positive regulation of endothelial cell proliferation			*
protein binding			
protein heterodimerization activity			
response to drug			

Transcription
Stress Response
Development
Signaling
Proliferation / Apoptosis
Other
